# Magnetic sculpture-like tumor cell vaccines enable targeted *in situ* immune activation and potent antitumor effects

**DOI:** 10.7150/thno.107162

**Published:** 2025-04-13

**Authors:** Heng Zhang, Qing-qing Li, Yue Shi, Lei Zhang, Kai-wen Wang, Ting Wu, Shan-bin Cheng, Zi-ren Zhang, Lu-ning Qin, Yun-long Zhao, Xue-ting Zhen, Hao-ran Ren, Lin-yong Du, Hui-juan Liu, Tao Sun

**Affiliations:** 1 State Key Laboratory of Medicinal Chemical Biology and College of Pharmacy, Nankai University, Tianjin, China.; 2 Tianjin International Joint Academy of Biomedicine, Tianjin, China.; 3 Key Laboratory of Laboratory Medicine, Ministry of Education of China, School of Laboratory Medicine and Life Science, Wenzhou Medical University, Wenzhou, Zhejiang, China.

**Keywords:** Immunotherapy, Whole-cell vaccines, Magnetic Sculpture-like cell.

## Abstract

**Rationale:** Tumor cells are ideal candidates for developing cancer vaccines due to their antigenic profiles, yet existing whole-cell vaccines lack efficacy. This study aimed to develop a novel whole-cell vaccine platform that combines immunogenicity, structural integrity, and tumor-targeting capabilities.

**Methods:** We created “Magnetic Sculpture-like (MASK) Cells” by treating tumor cells with high-concentration FeCl_3_, inducing rapid morphological fixation without traditional chemical crosslinking. MASK cells were characterized for proliferative capacity, biomolecule retention, and magnetic properties. Vaccine efficacy was tested *in vitro*, in melanoma-bearing mouse models, and through spatial transcriptomic profiling of tumor microenvironments. Combination therapy with anti-PD-1 was further evaluated.

**Results:** MASK cells lose proliferative ability but retain biomolecules and architecture. MASK cells promote dendritic cell maturation and T cell responses against tumors. Vaccines combining MASK cells and adjuvant potently suppress melanoma growth. Uniquely, FeCl_3_ sculpting imparts magnetism to cells, enabling directional navigation to tumors using magnetic fields and enhanced *in situ* immune activation. Spatial transcriptomics reveals DC and T cell activation and tumor cytotoxicity after MASK vaccination. Combined with anti-PD-1, MASK cell vaccines strongly inhibit growth and improve survival.

**Conclusion:** MASK cells represent a promising new approach for targeted, patient-specific anti-tumor therapeutics.

## Introduction

Cancer vaccines represent a promising immunotherapy strategy that harnesses the patient's adaptive immune system to combat malignant tumors. These vaccines work by stimulating immune responses against specific tumor antigens, with the key requirement being the efficient delivery of high-quality antigens to dendritic cells (DCs) for optimal activation 1-4. However, current limitations in sequencing and bioinformatics technology restrict our ability to identify and quantify tumor-specific antigens in individual samples 5, 6, hampering the development of timely personalized treatments.

Whole tumor cell vaccines have emerged as a compelling solution to this challenge. Since autologous tumor cells naturally carry diverse tumor-specific antigens, they serve as an ideal antigen source for cancer vaccine preparation 7-9. These techniques have demonstrated success in generating robust antitumor immune responses across various cancer types 9-15. However, existing methods face significant limitations, particularly in terms of stimulating tumor immunogenicity 16 and achieving precise targeting to avoid damage to normal cells 17. These challenges have restricted their clinical application 18, 19, highlighting the need for innovative therapeutic strategies.

Immune checkpoint blockade (ICB) drugs represent another significant advancement in cancer immunotherapy, achieving remarkable success in clinical practice. While ICB treatments have revolutionized cancer care and improved remission rates 1, their effectiveness varies considerably across cancer types, and many patients show resistance to these treatments 20, 21. This variable response has led to the exploration of combination therapies. The integration of therapeutic whole tumor cell vaccines with ICB drugs presents a particularly promising approach, leveraging both tumor antigen specificity and immune checkpoint inhibition to potentially enhance overall response rates and achieve more durable therapeutic outcomes 1, 22.

In our investigation of novel vaccine approaches, we discovered an unexpected phenomenon when treating tumor cells with 100 mM FeCl_3_ solution. Under phase contrast microscopy, we observed distinct morphological changes, characterized by enhanced contrast between cell and nuclear membranes. The cells underwent a transformation within approximately 30 s, assuming a distinctive sculpture-like appearance. While this process shares similarities with cell fixation, it differs significantly from conventional paraformaldehyde (PFA) fixation, leading us to designate these transformed cells as Sculpture-Like Cells (SLCs). Further investigation revealed that these cells possess magnetic properties, enabling their concentration within solid tumors under magnetic field guidance. This capability to effectively activate localized immune responses in tumor regions led to their alternative designation as Magnetic Sculpture-like (MASK) cells. These MASK cells present a potential solution to the limited response rates observed with existing tumor vaccines and show promise as a novel targeted, personalized therapeutic vaccine. Moreover, their potential synergy with ICB drugs suggests opportunities for enhanced antitumor effects through combination therapy.

## Methods

### Animals

Four to six-week-old male and female C57BL/6, BALB/c mice were purchased from Beijing Vital River Laboratory Animal Technology Co., Ltd. and acclimatized for one week in the specific pathogen-free facilities before performing experiments. They were kept in individually ventilated cages (IVC) with a controlled temperature (maintained at 22 ± 2 °C), relative humidity (kept at 40 - 60%), and a 12-h light/dark cycle. All of the experimental protocols have been approved by the Ethics Committee of Nankai University, approval number: 2022-SYDWLL-000589. Once the tumors were palpable, the mice were randomly assigned to different experimental groups. Randomization was carried out using a computer-generated randomization schedule to ensure unbiased allocation of animals to each group. Each group consisted of 6 mice.

### Cell culture

PLC-PRF-5, A549, MCF-7, HCT-116 and HUVEC cell lines were bought from the KeyGen Biotech (Nanjing, China). B16-F10-luciferase, B16-F10-GFP, H22- luciferase, MC38 and B16F10-OVA cell lines were bought from Shanghai Zhong Qiao Xin Zhou Biotechnology, cultured in DMEM or 1640 medium supplemented with 10% fetal bovine serum (Biological Industries) and 1% penicillin-streptomycin solution (KeyGen) in a humidified environment containing 5% CO_2_ at 37 °C. All cell lines were subjected to STR analysis, and the cell line mycoplasma-free status was verified using the MycoAlert mycoplasma detection kit (Lonza, LT07-218).

### Preparation of SLCs and SLCV (MASKv) for immunotherapy

For the preparation of SLCs, 1 × 10^7^ B16F10-luciferase cells were collected by centrifugation for 5 min at 800 rpm, and the cells were resuspended in 1 mL of PBS solution containing 100 mM FeCl_3_, treated for 5 min, and then centrifuged to collect the cells and washed three times with sterile PBS solution. Finally, SLCs were collected by centrifugation and resuspended in PBS and stored at 4 °C for use. To prepare SLCV (MASKv), one dose requires mixing 1×10^6^ SLCs with 20 μg of MPLA (Invitrogen, tlrl-mpla).

### Cell imaging

PLC-PRF-5, A549, MCF-7, HCT-116 and HUVEC cells were treated with PBS, 4% paraformaldehyde or 100 mM FeCl_3_, respective. Bright field and phase contrast microscopy images were then taken using a Nikon confocal microscope. The picture in the red dotted box in the phase contrast microscope image is enlarged for subsequent analysis. The intensity changes in the red dashed area were analyzed by NIS-Viewer software 5.21.00. For Live cell imaging, 3 × 10^6^ PLC-PRF-5 cells were inoculated into 24-well glass culture dishes (NEST). After culturing for 12 h, freshly prepared 100 mM FeCl_3_ solution was added to the well plate, and live cell imaging was performed immediately under the phase contrast microscope, and the during time was 1 min, and the time interval was 10 s.

### Scanning electron microscopy

For scanning electron microscopy, PLC-PRF-5 cells were seeded in 24-well plates containing slides, and after treatment with PBS with or without FeCl_3_, samples were fixed with 2.5% glutaraldehyde overnight at 4 °C. After washing three times with PBS, the samples were dried and dehydrated with graded ethanol and graded tert-butanol. The samples were concentrated at 4 °C and vacuum-dried overnight. The samples were sprayed with gold and then observed with a scanning electron microscope (SEM, COXEM).

### Immunofluorescence staining

Cells were cultured on coverslips in 24-well plates, and then were washed three times with PBS, fixed with 4% paraformaldehyde for 20 min and permeabilized with blocking buffer (QuickBlock Blocking Buffer, Beyotime) for 30 min at room temperature. Subsequently, the cells were incubated with primary antibodies VEGFR-2 (Abcam, ab10972), beta Tublin (Affinity, T0023), GAPDH (Affinity, AF7021) for 2 h at room temperature. The cells were washed with PBS (3 × 5 min) and incubated with fluorochrome labeled secondary antibody for 2 h at room temperature. Finally, coverslips were stained with DAPI and the analysis of immunofluorescence using ZEISS LSM 800 Inverted Confocal microscope attached with an Airyscan area detector.

### Proteomic analysis

The proteomic technology provided by Novogene Bioinformatics Technology Co., Ltd. (Beijing, China) was employed to comprehensively characterize the protein expression profiles of B16F10 cells before and after treatment with 100 mM FeCl_3_. Briefly, total proteins were extracted from the samples, quantified, and then subjected to enzymatic digestion and desalting. The resulting peptides were analyzed by liquid chromatography-tandem mass spectrometry (LC-MS/MS) in data-independent acquisition (DIA) mode. Raw mass spectrometry data were processed and searched using DIA-NN software (Direct DIA) against the UniProt database (Mus_musculus_uniprot_2024_07_26_Swissprot.fasta, 17,224 sequences), with a false discovery rate (FDR) set to 1%. Differentially expressed proteins were identified based on a fold change threshold of > 2.0 or < 0.5 and a *P*-value < 0.05, as determined by Student's t-test.

### Cell viability and apoptosis assay

The cell viability of SLCs was evaluated by Calcein/PI Cell Viability Assay Kit (Beyotime, C2015M), the staining protocol followed the manufacturer's instructions. Green fluorescent calcein-AM and red fluorescent propidium iodide (PI) were used to indicate live and dead cells, respectively, and stained cells were imaged using a confocal microscope with a 488 nm laser and a 550 nm laser.

For apoptosis analysis, SLCs were stained using the Annexin V-FITC Apoptosis Detection Kit (Beyotime, C1062L) and analyzed by flow cytometry (BD LSRFortessa) after incubation for 30 min at 4 °C.

### Cell proliferation assay

Cell proliferation ability was assessed by Cell Counting Kit-8 (APExBIO, K1018). According to the manufacturer's instructions, at 0, 24, 48 and 72 h after PLC-PRF-5 cells treated with FeCl_3_, 10 μL of CCK8 solution was added to each well, and incubated at 37 °C for 2 h. The absorbance of each well was measured at a wavelength of 450 nm using a microplate reader (Thermo Fisher).

### Wound healing assay

For wound healing experiments, PLC-PRF-5 cells were seeded in 24-well plates and cultured. After the cells developed a 90% confluent monolayer, use a sterile pipette tip to remove the cells evenly, and continue to culture the cells in serum-free medium after treatment with or without FeCl_3_ solution. At 0, 12, 24, and 48 h, the scratch field of view was selected under the microscope, and the cell migration was photographed and measured. Wound healing rate was analyzed by Image J software.

### Gelatin degradation assay

To assess the invasive ability of SLCs, we performed a fluorescent gelatin substrate degradation assay. Porcine skin gelatin (Thermo Fisher, G13187) diluted with 2% sucrose was used at a final concentration of 0.2 mg/mL. The PLC-PRF-5 cells were inoculated on gelatin-covered 24-well slides, and after culturing for 6 h, 100 mM FeCl_3_ was added to treat the cells. After continuing to culture for 24 h, the control cells and SLCs were fixed with 4% paraformaldehyde, and the cytoskeleton was labeled with YF 633-Phalloidin (US Everbright, YP0053S) and mounted with DAPI. Finally, images were acquired using a Zeiss LSM 800 inverted confocal microscope. Gelatin degradation was analyzed using Image J software.

### *In vivo* cell proliferation of SLCs

To verify whether SLCs still have the ability to proliferate in mice, 3 × 10^6^ SLCs or live B16F10-luciferase cells were inoculated subcutaneously into mice, and the bioluminescence signal was monitored on day 18.

### Atomic force microscopy

The DMT modulus of the sample was measured using a Bruker Dimension Icon atomic force microscope operated in PeakForce Tapping mode. Prior to imaging, the SCANASYST-AIR probe (length: 115 µm, width: 25 µm, resonant frequency: 70 kHz, spring constant: 0.4 N/m, Bruker) was calibrated using the thermal tune method to determine the precise deflection sensitivity and spring constant. Scanning was performed in air at room temperature, with a scan rate of 0.7 Hz and a peak force setpoint of 8 nN. In the Channels section, the parameters Height, DMT Modulus, and Adhesion were selected for simultaneous mapping.

### Bioluminescent imaging

The IVIS imaging system (Perkin Elmer) was used for bioluminescence imaging studies in mice. D-Luciferin potassium (150 μg/g, meilunstar, 115144-35-9) was injected intraperitoneally into the mice, and the mice were anesthetized when the peak fluorescein uptake was reached 10 min after the injection, and then imaged, and the bioluminescent intensity was calculated using Living Image software.

### Isolation and activation of BMDCs

To collect BMDCs, 7-week-old C57BL/6 mice were euthanized, sterilized by immersion in 75% ethanol, and the tibiae and femurs were removed and the bones were rinsed with PBS to obtain bone marrow cells. After centrifugation and collection, the bone marrow cells were cultured in RPMI-1640 medium (10% FBS) containing 20 ng/mL granulocyte/macrophage colony-stimulating factor (GM-CSF, Abclonal, RP01206) and 10 ng/mL IL4 (Abclonal, RP01161). On day 3, the supernatant is gently removed and the same fresh medium was added. On day 7, BMDCs were collected and spread evenly in 6-well plates at a density of 1 × 10^6^ cells, with the experimental group adding 1 × 10^6^ SLCs-treated BMDCs and the wells without SLCs serving as the control group. After 48 h of treatment, BMDCs were collected and cells were stained with antibodies CD40 (PTMa, PTM-5537), CD80 (PTMa, PTM-5247), CD86 (PTMa, PTM-5570) and MHC II (Invitrogen, 14-5321-82), respectively. The secondary antibody CoraLite 488-conjugated Goat Anti-Mouse IgG(H+L) (Proteintech, SA00013-1) or Rhodamine (TRITC)-conjugated-Goat Anti-Rat IgG(H+L) (Proteintech, SA00007-7) was used, and analyzed using flow cytometry.

### *In vitro* tumor killing assay

CD8^+^ T cells were isolated and purified from the spleen of 7-week-old C57BL/6 mice using the CD8 T Cell Isolation Kit (Biolegend, 480008). 1 × 10^6^ B16F10-GFP cells were inoculated in six-well plates. CD8⁺ T cells were added to bone marrow-derived dendritic cells (BMDCs) and co-cultured with B16F10-GFP cells (CD8⁺ T cells: BMDCs: B16F10-GFP cells = 2: 1: 2). Additionally, BMDCs and CD8⁺ T cells were separately co-cultured with B16F10-GFP cells at ratios of (BMDCs: B16F10-GFP cells = 1: 2) and (CD8⁺ T cells: B16F10-GFP cells = 1 : 1), respectively. Experimental groups were established by adding specific numbers of SLC cells (1 × 10^5^ or 5 × 10^5^), along with control groups without SLC cells. All co-culture systems were incubated for 48 h. Finally, the viability of B16F10-GFP cells was analyzed by flow cytometry.

### Detection of Tumor Antigen-Specific T Cells

B16F10 cells (3 × 10^6^) were subcutaneously injected into the right flank of mice. The mice were then randomly divided into four groups. On days 7, 10, and 13 post-tumor inoculation, the four groups were intravenously (i.v.) administered phosphate-buffered saline (PBS), 1 × 10^6^ SLCs, adjuvant (monophosphoryl lipid A, MPLA, 20 μg/mouse, Invitrogen, Cat# tlrl-mpla), or SLCV (a combination of MPLA and SLCs), respectively (n = 6). On day 16 post-tumor inoculation, splenocytes were collected, and tumor antigen-specific T cells were quantified using the B16F10 tumor antigen-specific T cell detection kit (ESMDT-MB16-1). The following antibodies were used for analysis: anti-mouse CD3 FITC (BioLegend, 100204), anti-mouse CD8a 605 (BioLegend, 100743), and anti-mouse IFN-γ PE (Proteintech, PE-65153).

### Subcutaneous tumor models and treatment

3×10^6^ B16F10-Luciferase cells were injected subcutaneously into the right side of female C57BL/6 mice to construct a melanoma tumor model, and the tumor dimensions of the mice were measured every two days using vernier calipers. Tumor volume (mm^3^) was calculated as length (mm) × width (mm)^ 2^ × 0.5. And bioluminescence imaging was performed on days 7, 12, and 18. For FeCl_3_ treatment, when the subcutaneous tumor volume of mice reached 100 mm^3^, 100 mM FeCl_3_ was injected *in situ* at multiple points around the tumor every 3 days for a total of 3 injections (n = 6). For SLCs treatment, 1 × 10^6^ SLCs were intravenously injected (i.v.) on days 7, 10, and 13 after the mice were subcutaneously inoculated with tumor cells, respectively (n = 6). For SLC vaccine treatment, mice were intravenously injected (i.v.) with PBS, 1×10^6^ SLCs, Adjuvant (MPLA, 20 μg/mouse, Invitrogen, tlrl-mpla), or SLCV (combined treatment with MPLA and SLCs) on days 7, 10, and 13 after tumor inoculation, respectively (n = 6). Unless otherwise specified, the control group was treated with PBS.

### Immune memory effect of SLC vaccine

To investigate the immune memory effect of SLC vaccine, mice treated with SLC vaccine were re-challenged. Briefly, 3 × 10^6^ B16F10-Luciferase cells were inoculated subcutaneously on the right side of the mice. When the tumor volume reaches 80-100 mm^3^, and the mice were intravenously injected (i.v.) with Adjuvant (20 μg/mouse) or SLC vaccine on days 7, 10, and 13 after tumor inoculation, respectively (n = 6), and the tumors were completely excised at day 16. Thirty days after the first tumor excision, 3 × 10^6^ B16F10-Luciferase cells were inoculated subcutaneously on the left side of the mice, and the tumor volume was recorded. When the tumor volume reached about 1800 mm^3^, the mice were euthanized and the tumors were isolated for flow cytometry analysis.

### Tumor-initiating assay

Tumor-initiating abilities were investigated by limiting dilution and serial transplantation assays. 4 to 6-week-old male and female C57BL/6 mice were first treated with adjuvant (MPLA), SLC or SLCV. Then they were injected subcutaneously with 1000, 10^4^ or 10^5^ B16F10 cells. Tumor incidence and tumor latency were recorded. Tumor-initiating frequency and P value were calculated using the Extreme Limiting Dilution Analysis (ELDA) software.

### Cytokine measurement by ELISA

After 18 days of SLCs treatment, the immune response in C57BL/6 mice was examined. Briefly, 300 μL of blood was collected by removing the eyeballs of mice, and after the blood was allowed to stand at room temperature for 30 min, it was centrifuged at 3000 rpm for 15 min to obtain the supernatant serum. Serum levels of IL-4 (proteintech, KE10010) and TNF-α (proteintech, KE10002) were determined using ELISA kits.

### Magnetic measurement of SLCs

The magnetic properties of the magnetic SLCs were measured using the equipment of the Physical Property Measurement System (PPMS-9) within a magnetic field strength range of ± 4e^4^ (oe).

### Magnetic targeting therapy of MASK vaccine

In order to investigate whether MASK vaccine with magnetic properties can exert a greater anti-tumor effect under the action of a magnetic field, the therapeutic effect was evaluated using subcutaneous tumor-bearing mice. First, 3 × 10^6^ B16F10-Luciferase cells were injected subcutaneously into the right side of the mice for 7 days, and the mice were randomly divided into four groups: PBS, Magnet, MASKv, and MASKv + Mag (n = 6). The mice were intravenously injected (i.v.) with PBS or MASKv (MASKs 1 × 10^6^ + Adjuvant 20 μg/mouse) on days 7, 10, and 13, respectively. Mice in the Magnet or MASKv + Mag groups were maintained for 1 day after each PBS or MASKv injection with a strong magnet bound to the tumor on the back of the mice. Tumor volumes were recorded every two days during treatment. After 19 days of treatment, tumor tissues were collected for HE staining, IHC staining, and flow cytometric analysis.

### Immunotherapy with MASK vaccine combined with α-PD-1

As previously described, C57BL/6 mice subcutaneously inoculated with B16F10-Luciferase cells were randomly divided into five groups: PBS, MASKv +M, α-PD-1, MASKv +α-PD-1 and MASKv +M+α-PD-1. On days 7, 10, and 13 after tumor inoculation, mice were intravenously injected (i.v.) with PBS or MASKv, and for the α-PD-1 treatment group, 200 μg α-PD-1 monoclonal antibody (Bio X Cel, BE0273) was used to treat tumor-bearing mice intraperitoneally (i.p.). Magnet processing group according to the previous method. Tumor volumes were monitored, and the tumor tissues were collected to analyze the immune response.

### Immunohistochemical analysis

Routine histological and immunohistochemical examinations were performed using tissue sections. Briefly, tumors from each group of mice, or organs of heart, liver, spleen, lung, and kidney from some groups of mice, were harvested after the experiment, fixed in 10% formalin, then paraffin-embedded, cut to 5 μm thickness, and fixed on slides. H&E (Solarbio, G1121) staining and a series of IHC stains were performed according to the instructions. For IHC staining, primary antibodies: Ki67 Rabbit PolyAb (proteintech, 27309-1-AP), Anti-CD8 alpha Mouse mAb (PTMa, PTM-5186), plus Polyer HRP (Mouse/Rabbit) IHC Kit (MXB biotechnologies, Kit-9902) and DAB peroxidase substrate kits (MXB biotechnologies, DAB-0031) were used. Finally, the slides were observed with a light microscope.

### Flow cytometry analysis

Tumor or spleen tissues from mice in each group were collected, the tumors were cut into small pieces with scissors, and digested with 1 mg/mL collagenase IV (Solarbio, C8160) at 37 °C for 1 h. The digested tissues were filtered through a 100 μm cell filter and collected to obtain a single cell suspension. Cells were fixed using 4% paraformaldehyde for 20 min, followed by staining with a series of fluorophore-coupled antibodies for 30 min at 4 °C in the dark. The following antibodies were used: Anti-mouse CD45 APC (Invitrogen, 17-0451-83), anti-mouse CD3 FITC (BioLegend, 100204), anti-mouse CD4 PE (Invitrogen, 12-0441-83), anti-mouse CD8 PE (sungene, M10083-09D), anti-mouse CD8a 605 (BioLegend, 100743), anti-mouse CD62L APC (Invitrogen, 17-0621-82), anti-Hu/Mo CD44 PE (Invitrogen, 12-0441-82), anti-mouse IFN-γ PE (Proteintech, PE-65153), anti-mouse TNF-α PE (BioLegend, 506305). All samples were collected on a flow cytometer (BD LSRFortessa) and data were analyzed by Flowjo software.

### Spatial transcriptomics sequencing

As previously described, C57BL/6 mice 7 days after subcutaneous inoculation with B16F10-luciferase cells were randomly divided into three groups: Control (PBS), MASKv D7 and MASKv D13, and each group applied an external magnetic field after each dose according to the previous method. MASKv D7 were treated with MASKv on days 1, 4; MASKv D13 were treated with MASKv on days 1, 4, 7, and 10, and all the tumor tissues were collected three days after the last treatment. Finally, freshly tumor models were performed according to the Spatial Transcriptomics protocol (10× Genomics Visium Cytassist). Briefly, after tumor tissues were fixed, HE stained, and observed by imaging, the tissues were permeabilized according to the 10× Spatial kit (10× Genomics, Pleasanton, CA). The mRNA of the permeabilized tissues were released and bound to the corresponding capture probe, followed by library preparation and sequencing on the Illumina platform, and finally data visualization analysis was performed based on the HE staining results.

### Analysis of spatial transcriptomics

The Space Ranger software pipeline (version 1.0.0) provided by 10×Genomics was used to process Visium spatial RNA-seq output and detect the brightfield microscopy images of tissue. We processed the unique molecular identifier (UMI) count matrix using the R package Seurat (R 4.1.3). We first normalized the data with sctransform in order to account for variance in sequencing depth across data points, detecting high-variance features, and stores the data in the SCT assay. Top variable genes across single cells were identified using the method described in Macosko et al. Briefly, the average expression and dispersion were calculated for each gene, genes were subsequently placed into x bins based on expression. Principal component analysis (PCA) was performed to reduce the dimensionality on the log transformed gene-barcode matrices of top variable genes. Cells were clustered based on a graph-based clustering approach, and were visualized in 2-dimension using tSNE. Likelihood ratio test (LRT) that simultaneously test for changes in mean expression and in the percentage of expressed cells was used to identify significantly differentially expressed genes between clusters. Differentially expressed genes (DEGs) were identified using the FindMarkers function of Seurat package. P value < 0.05 and |log2foldchange| > 0.5 was set as the threshold for significantly differential expression. GO enrichment and KEGG pathway enrichment analysis of DEGs were respectively performed using R based on the hypergeometric distribution.

### Quantification and statistical analysis

Statistical analyses were performed using GraphPad Prism version 9 for Windows or R 4.1.3. Statistically significant differences were calculated using two-tailed unpaired t-tests, or unpaired t test with Welch's correction, Pearson's correlation, and Kaplan-Meier as needed. *P* < 0.05 was considered significant.

## Results

### High concentrations of FeCl_3_ can induce cell sculpture-like death *in vitro*

In an experiment to form Prussian blue nanozyme *in situ* in cells, FeCl_3_ and K_4_[Fe(CN)_6_]-3H_2_O need to be added sequentially to the cells. When preparing various concentrations of FeCl_3_ solutions, an increase in the concentration resulted in an escalation of precipitates in complete medium, DMEM+ Fetal Bovine Serum (FBS). This occurrence may be linked to the salting out of proteins in FBS. It is noteworthy that there was almost no precipitate left in the solution once the FeCl_3_ concentration reached 100 mM (Figure S1A, shown in red box). Continued increases in the concentration of FeCl_3_ also failed to yield a significant precipitate, albeit the solution's absorbance increases steadily. This phenomenon was not observed in PBS or empty medium (DMEM), serving as a control. In the complete medium, the absorbance of FeCl_3_ exhibited a noticeable dip at the 100 mM concentration (indicated by the arrow in Figure S1B), indicating inhibition of protein salting-out. We hypothesized the presence of an “antisalting effect” in this solution, which may have unique implications. Accordingly, we added 100 mM FeCl_3_ to the cells for observation. Upon the solution's addition, a morphology distinct from that of viable cells and 4% PFA fixation promptly manifested under the phase contrast microscope.

**Figure 1:**
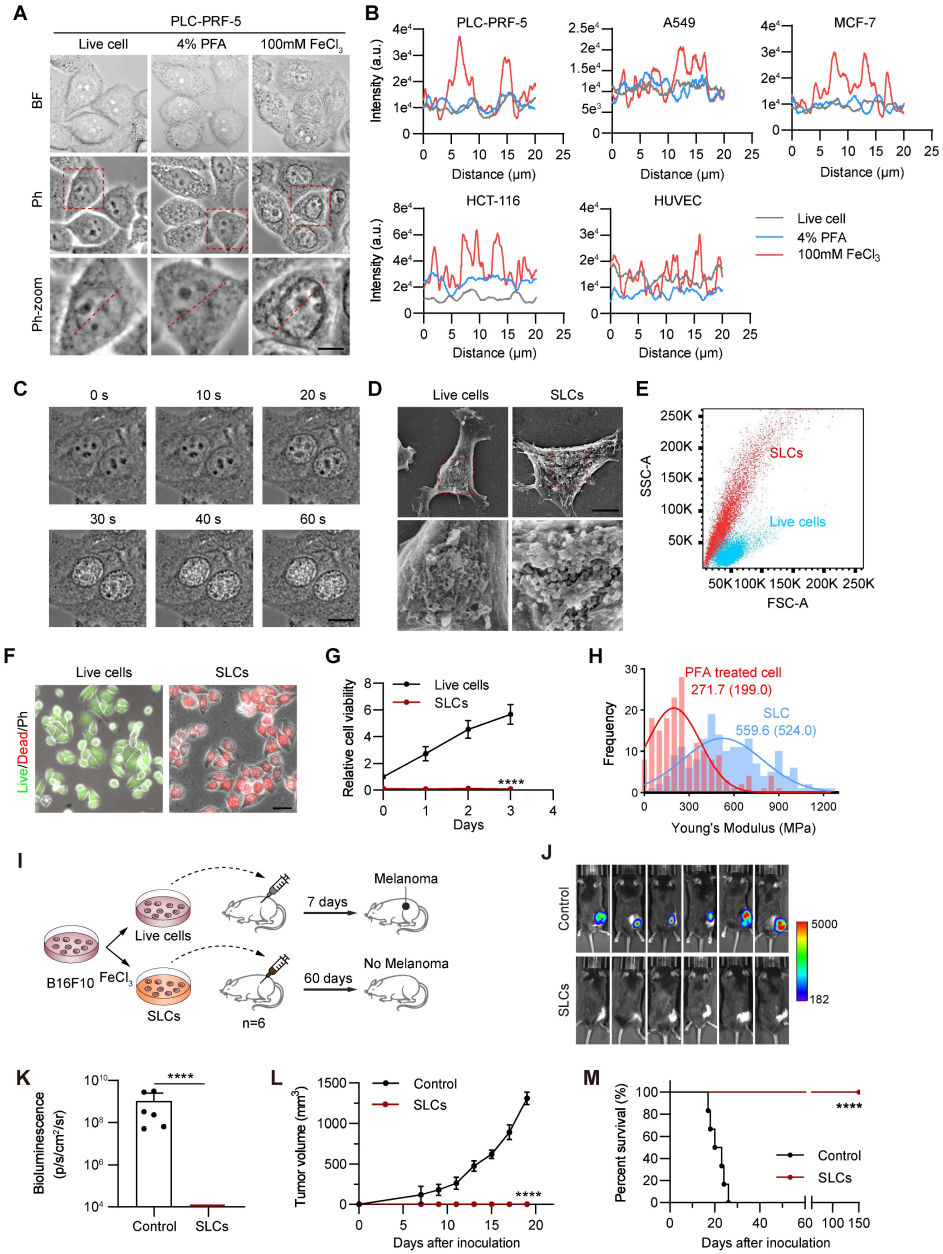
** Characterization of Sculpture-Like Cells after treatment with 100 mM FeCl_3_. (A)** Representative bright field (top) and phase contrast (middle) microscopy images of PLC-PRF-5 cells treated with PBS, 4% paraformaldehyde, or 100 mM FeCl_3_. The red dashed box represents a magnified view of the phase contrast microscopy image. Scale bar, 5 µm. **(B)** Density plot of the red dashed area in the magnified image of phase contrast images in (A) and four additional cell lines.** (C)** High speed live cell imaging of PLC-PRF-5 cells treated with 100 mM FeCl_3_ under a phase contrast microscope. The time interval between each picture is 20 s. Scale bar, 10 µm.** (D)** Scanning electron microscopy (SEM) images of PLC-PRF-5 live cell or cell after treatment with FeCl_3_. Scale bar, 5 μm. **(E)** Flow cytometric analysis of PLC-PRF-5 live cells and cells treated with FeCl_3_. FSC, forward scatter; SSC, side scatter. **(F)** Cell viability analysis of living cells and cells treated with FeCl_3_ was performed using the Calcein/PI Cell Viability kit. Calcein AM: living cells; PI (propidium iodide): dead cells. Scale bar, 30 μm. **(G)** Relative cell viability (%) analysis of live cells and FeCl_3_ treated cells (n = 3) by CCK8 assay. **(H)** Histograms and Gaussian fitting line of the Young's modulus of PFA treated cell and SLC (n = 3, collected point = 174). The values represent the mean value of cell's elasticity, and the values in parentheses represent the highest values of the Gaussian fitting line.** (I)** Schematic diagram of experimental design.** (J-K)** Representative images of bioluminescence signal **(J)** showing *in vivo* proliferation of luciferase-labeled 3×10^6^ B16F10 viable cells and SLCs in C57BL/6 mice after inoculation on day 19 (n = 6) and quantification of bioluminescence signal (p/s/cm^2^/sr) is shown** (K)**, n = 6, biological replicates. **(L)** Tumor volume of mice after challenge with live cells or SLCs (n = 6). **(M)** Kaplan--Meier survival curves of the mice of different treatment groups (n = 6). Data represent means ± S.D, and were analyzed by two-tailed unpaired t tests with GraphPad Prism software, ****P < 0.0001.

As shown in Figure 1A, using the typical epithelial-like cells PLC-PRF-5 as an example, bright field and phase contrast microscopy images of live cells, cells fixed with PFA and cells treated with 100 mM FeCl_3_ are shown. Under phase contrast microscopy, it can be observed that after treatment with 100 mM FeCl_3_, the boundaries between the cell membrane and the nuclear membrane become clearer, the interior of the cell becomes translucent, and the cells, especially the nucleus, have a more granular appearance. We repeated this experiment on lung cancer cells (A549), breast cancer cells (MCF-7), colon cancer cells (HCT-116) and normal endothelial cells (HUVEC) and found that these cells all showed similar characteristics after the addition of 100 mM FeCl_3_ (Figure S1C). This may be a new mode of death that does not rely on biological processes. Because the shape resembled a sculpture, we named them “Sculpture-Like Cells” (SLCs). Statistics of the optical density of the nucleus, nuclear membrane, cytoplasm, and cell membrane of living cells, PFA fixed, and 100 mM FeCl_3_-treated cells were observed under a phase contrast microscope. As shown in Figure 1B, compared with live cells and cells fixed with 4% PFA, the optical density characteristics of cells treated with 100 mM FeCl_3_ are significantly different and the fluctuation range becomes larger. This is the characteristic that can be identified by SLCs. To better observe the formation process of SLCs, we took PLC-PRF-5 as an example and performed high-speed live cell imaging (Figure 1C and Video S1), which can clearly show that after FeCl_3_ addition for 20 s, the cells immediately showed sculpture-like characteristics. To investigate whether the formation of SLC is specific to ferric chloride, we treated the cells with various metal salts at the same concentration. After treating the cells with ferric sulfate, we observed the same SLC formation as with FeCl_3_ treatment. When treated with equimolar concentrations of FeCl_2_, CuCl_2_, AlCl_3_, and ZnCl_2_, the cells exhibited some morphological changes, but these changes were distinct from SLC (Figure S1D). This indicates that the formation of SLC is primarily associated with trivalent iron ions and is independent of the type of anion.

We further observed SLCs with a scanning electron microscope. As shown in Figure 1D, the cell structure of SLCs is intact, but the surface is rougher. Compared with untreated live cells, there are a large number of protrusions on the surface. By analyzing the forward angle (FSC) and lateral angle (SSC) of cells by flow cytometry, it can be found that compared with normal fixed cells, the FSC of SLCs has no obvious change, but the SSC increases significantly (Figure 1E), indicating that the granularity of SLCs increased, which was consistent with the scanning electron microscopy images. We performed transmission electron microscopy (TEM) analysis on both normal fixed cells and SLCs. As evidenced in Figure S1E, compared to normal cells, FeCl_3_-treated cells exhibited preserved overall morphology, with intact cell membranes and clearly discernible nuclear envelopes. Furthermore, chromatin condensation is evident, and the cytoplasm displays numerous vacuoles, indicative of cellular stress. These findings align with the biochemical perturbations caused by FeCl_3_ hydrolysis, which generates localized acidity and elevated ionic strength. We further investigated whether SLCs also preserve proteins, DNA and other molecules. The proteins of living cells and SLCs were extracted, subjected to SDS-PAGE, and then stained with coomassie brilliant blue. Figure S1F shows that there is no significant difference in the protein bands obtained between the two groups. By immunofluorescence and DAPI staining of cytoskeleton β-tubulin, cell metabolic enzyme GAPDH and receptor VEGFR2 (Figure S1G), it can be seen that DNA and three proteins can be detected in SLCs, and there was no significant difference from the normal cell group. These results indicate that SLCs essentially retain DNA and proteins after FeCl_3_ treatment.

We conducted proteomic analyses on normal cells and SLC. As illustrated in Figure S1H, protein expression profiles between the two groups were highly correlated, with an *R*^2^ of 0.93, indicating nearly identical expression levels. The volcano plot in Figure S1I further shows that the vast majority of proteins did not exhibit significant differential expression, with only 43 proteins showing changes. These differentially expressed proteins were not enriched in programmed cell death pathways but instead appeared to reflect basic cellular perturbations. Additionally, hierarchical clustering of known programmed cell death markers (including apoptosis, pyroptosis, ferroptosis, and necroptosis) in SLC and control viable cells (Figure S1J) revealed virtually no differences in expression levels.

### SLCs have no cell viability and tumorigenic ability

One week after the formation of SLCs, we noticed that they did not proliferate significantly, so we further investigated whether the SLCs had died. Live/dead staining results showed that the cells in the SLCs group all had dead cell signals and no live cell signals (Figure 1F). CCK8 kit was used to detect mitochondrial activity, and the results showed that SLCs had almost no mitochondrial activity (Figure 1G). AnnexinV/PI double staining flow cytometry analysis also showed that more than 70% of SLCs were located in the Annexin V/PI double positive area, and more than 99% were located in the Annexin V positive area (Figure S1K). The above results indicate that SLCs are completely dead and have no cellular activity. Using the classic oncology wound healing assay and fluorescent gelatin degradation experiment, we also saw that SLCs lacked the ability to migrate and invade (Figure S1L-M). Additionally, we found that SLCs have a higher stiffness (evaluated by Young's modulus) compared to PFA-fixed cells, which may be a unique feature of SLC (Figure 1H).

We further designed animal experiments, as shown in Figure 1I, to investigate whether SLCs have tumor-forming ability *in vivo*. We used the C57BL/6 mouse subcutaneous transplantation tumor model, and subcutaneously inoculated the mice with luciferase-labeled B16F10 cells (Control) and B16F10 SLCs induced by FeCl_3_ *in vitro*. The results showed that after inoculation of B16F10 cells in the control group, it grew rapidly under the skin of mice (Figure 1J-L). After 19 days, the tumor volume reached the upper limit of animal ethics and the mice were euthanized (Figure S1N-O). All mice inoculated with SLCs were still alive 150 days later (Figure 1M), and no tumor formation was observed, indicating that SLCs do not have the ability to form tumors *in vivo*.

To further demonstrate that high concentrations of FeCl_3_-induced cell death are not a form of programmed cell death, we performed Western blotting to detect apoptotic markers (Cleaved Caspase-3, PARP1), necroptosis markers (pMLKL), and ferroptosis markers (GPX4 and xCT) in SLCs. As shown in Figure S1P-Q, the expression of these markers in SLCs was consistent with that in live cells, indicating that FeCl_3_-induced cell death is not any of these three major types of programmed cell death. Considering these characteristics, we believe that the process is not a form of programmed cell death.

### High concentrations of FeCl_3_ induce the sculpture-like death of tumor cells in the body, limiting tumor growth, but needs to be improved

The results of cell experiments show that 100 mM FeCl_3_ can cause cell death, loss of cell viability and tumorigenic ability. Therefore, multiple injections of 100 mM FeCl_3_ into the tumor may be a way to limit tumor growth. We designed the experiment as shown in Figure S2A to establish a luciferase-labeled B16F10 subcutaneous xenograft tumor model in C57BL/6 mice. Tumor formation was observed on day 7. Thereafter, 100 mM FeCl_3_ was injected into the tumor at multiple sites every 3 days for a total of 3 injections, and tumor growth was monitored. When the tumor reached the upper limit of animal ethics requirements, that is, on day 19 after tumor bearing, the mice were sacrificed. *In vivo* animal imaging results (Figure S2B) showed that mice injected with FeCl_3_ at multiple points in the tumor had lower B16F10 luciferase signals and smaller signal areas compared to the control group. We quantified the bioluminescence signals obtained by *in vivo* imaging (Figure S2C) and calculated the measured tumor volumes (Figure S2D). It can be seen that tumors injected with FeCl_3_ at multiple points in the tumor have a significantly lower growth rate than the control group. On day 19, the mouse tumors were isolated and weighed. It can be seen that the weight of the tumors injected with FeCl_3_ at multiple points *in situ* was also significantly lower than that of the control group (Figure S2E). The above results show that the injection of 100 mM FeCl_3_ into the tumor can significantly inhibit tumor growth.

However, FeCl_3_ is a strong electrolyte, and a concentration of 100 mM is relatively high. The blood in animals is essentially a sol in liquid state. Consequently, when a large amount of FeCl_3_ solution comes into contact with blood, rapid coagulation will occur, and the generated precipitates are likely to cause blockages. Therefore, it is widely used as a hemostatic agent in clinical practice and is also a classic reagent in the field of thrombosis research 23.

Considering that FeCl_3_ has certain harmful effects in the body, we next evaluated the safety of intratumoral injection of high concentration FeCl_3_. By observing the living conditions of the mice, we found that the mice in the intratumoral injection group of FeCl_3_ had dull hair color, were listless, and ate less. We also made gross and pathological observations on various organs of the mice. Although there was no significant difference in the weight of various organs of mice in the intratumoral FeCl_3_ injection group compared with tumor-bearing mice (Figure S2F), differences in color were observed in the liver. The livers of mice in the control group were reddish, whereas the livers of mice in the FeCl_3_ injection group were pale yellow (Figure S2G). The combination of FeCl_3_ and blood causes clotting 23. We believe that the mice in the intratumoral FeCl_3_ injection group may have a light yellow color due to the obstruction of blood circulation and the failure of the liver to receive sufficient blood infiltration.

In addition, hematoxylin and eosin (HE) staining results of various organs showed that the degree of inflammatory cell infiltration increased in the liver and spleen of mice in the intratumoral FeCl_3_ injection group (Figure S2G-K). HE staining results in the tumor area showed that after intratumoral injection of FeCl_3_, large areas of necrosis and inflammatory infiltration occurred, and there were SLCs with obvious characteristics such as brightened nuclei and increased cell granularity near the necrotic area and inflammatory infiltration area (Figure S2L), which have consistent characteristics with SLCs induced *in vitro*. The above results indicate that direct intratumoral injection of high concentration FeCl_3_ may have safety issues, but this treatment method can significantly enhance the immune response. Therefore, we further investigated the possibility of using SLCs obtained by pretreatment with high concentration FeCl_3_ as immunotherapy.

### Intravenous injection of SLCs can activate the immune system *in vivo* and treat tumors

**Figure 2:**
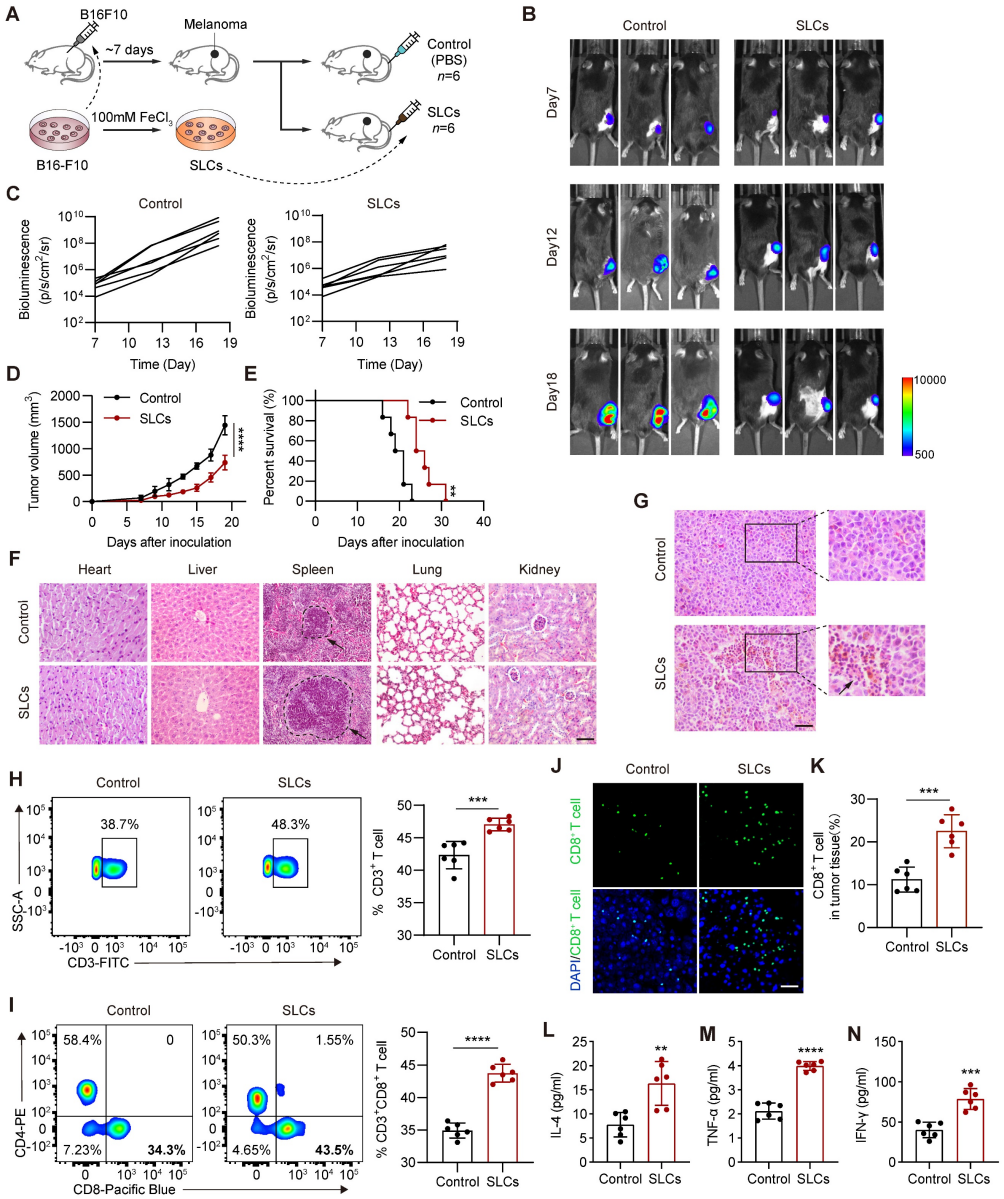
** SLCs activate immunity *in vivo* and exert anti tumor effects. (A)** Schematic of model construction and *in vivo* treatment of SLCs. Seven days after C57BL/6 mice were subcutaneously inoculated with tumor cells, SLCs were injected intravenously (i.v.) once every three days for a total of three times. Organ and tumor tissues were collected on day 19 to analyze immune responses.** (B)** Representative bioluminescence images and quantitative bioluminescence** (C)** of mice in different treatment groups (n = 6). **(D)** Tumor volumes were recorded every two days until day 19 (n = 6). **(E)** Kaplan--Meier survival curves of the mice of different treatment groups (n = 6). **(F)** HE staining of hearts, livers, spleens, lungs and kidneys. Scale bar, 100 µm. **(G)** HE staining of tumor tissue. Scale bar, 50 µm. **(H-I)** Representative flow cytometry data for frequency (left) and quantification (right) of tumor infiltrating CD3^+^ T cells **(H)** or CD3^+^ CD8^+^ T cells **(I)** (n = 6). **(J-K)** Immunofluorescence staining of CD8^+^ cells (green) in tumor tissue collected on day 19.** (J)** and quantification of CD8^+^ cells per field of view (n = 6) **(K)**. Scale bar, 30 μm. **(L-N)** Serum samples were isolated on day 19 and cytokine levels IL-4 **(L)**, TNF-α** (M)**, IFN-γ **(N)** were determined by ELISA assay (n = 6). Data represent analyses of the indicated n mice per group, means ± S.D, and were analyzed by two-tailed unpaired t tests with GraphPad Prism software. **P < 0.01; ***P < 0.001; ****P < 0.0001.

As shown in Figure 2A, we pretreated B16F10 cells with high concentration of FeCl_3_ *in vitro* to obtain SLCs. Although the SLCs were repeatedly washed with PBS during preparation, we were still concerned about the potential presence of high concentrations of iron ions. Therefore, we measured the iron content in the SLCs. We found that the average iron content of SLC was 0.771 ng per 10^4^ cells, while the average iron content of live cells was also 0.731 ng per 10^4^ cells. The iron content of SLC was only 1.05 times that of live cells, indicating that this concentration of iron is within the normal range (Figure S3A). Additionally, we measured the iron content of the SLC eluate. After placing 8 × 10^6^ SLC in 2 mL of PBS for one day, the iron content of the supernatant was found to be 0.08 μg/mL, which is below the national standard for iron content in drinking water (0.2 mg/L). Therefore, we believe that SLC does not have iron-related toxic side effects. To investigate the antitumor effect of SLCs *in vivo*, we subcutaneously inoculated luciferase-labeled B16F10 cells into C57BL/6 mice and injected SLCs into the tail vein on days 3, 6, and 9 after tumor formation. The control group was injected intravenously with equal volume of PBS. *In vivo* imaging (Figure 2B-C) and tumor growth curve (Figure 2D) showed that the tumor growth of mice in the tail vein injection group of SLCs was significantly inhibited and the survival time was also significantly prolonged (Figure 2E). Various organ tissues were harvested from the mice (Figure S3B-F), and HE staining was performed (Figure 2F). The results showed that mice in the SLCs injection group had no obvious damage to various organs, indicating that SLCs in mice have no significant side effects in the body. It is worth noting that the spleen, as the largest peripheral immune organ, plays an important role in the immune response of the human body 24. In the spleen tissues of mice in the SLCs group, we observed that the size and number of splenocytes were significantly increased and their structure was intact. HE staining of the tumor was then performed. The results are shown in (Figure 2G), compared with the control group, the degree of tumor immune infiltration was significantly increased in the SLCs group. The above results indicate that SLCs can effectively stimulate immunity *in vivo*.

T cells play an important role in antitumor immunity and are the major cell type that kills tumor cells and inhibits tumor progression. CD8^+^ T cells are the end effectors of cancer immune responses 25, 26, so we detected them by flow cytometry. As shown in Figure 2H-I, the proportion of infiltrating CD3^+^ T cells and CD8^+^ T cells in the tumor tissue of the SLCs group was significantly increased compared with the control group. Immunofluorescence staining of CD8^+^ T cells was performed on tissue sections from the tumor. As shown in Figure 2J, the proportion of CD8^+^ T cells, identified by CD8 staining, was significantly increased in the tumor tissue of the SLCs group compared to the control group (Figure 2K), indicating that SLCs primarily promote the infiltration of CD8^+^ T cells into tumors. To further assess T cell activation, we measured the levels of three key cytokines, IL-4 (Figure 2L), TNF-α (Figure 2M), and IFN-γ (Figure 2N). The results demonstrated a significant increase in the levels of these cytokines in the SLCs group compared to the control group, suggesting that SLCs can effectively activate T cells.

### SLCs stimulate DC maturation and differentiation *in vitro* and promote T cell killing of B16F10 cells

**Figure 3:**
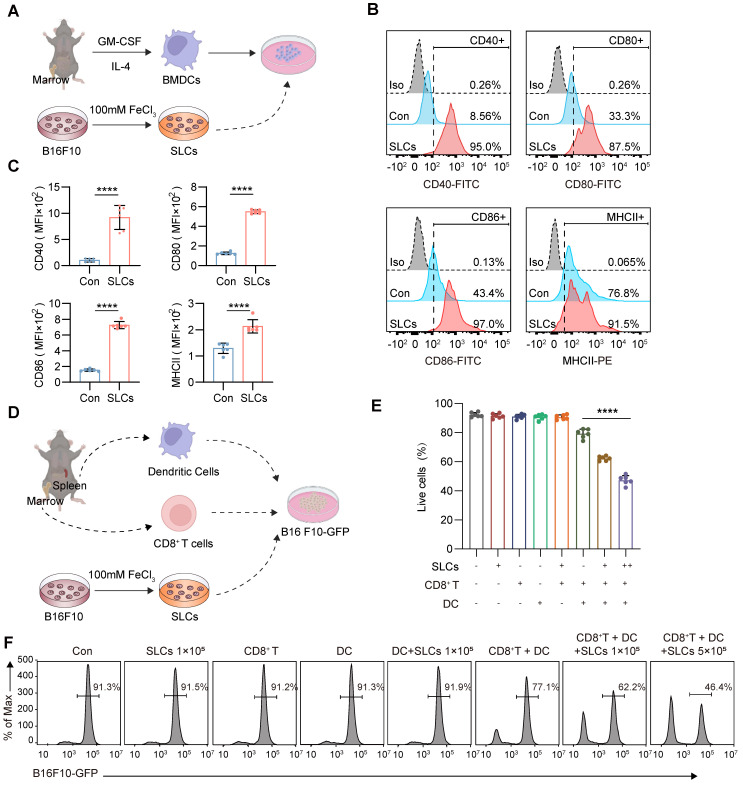
** SLCs promote DC maturation, differentiation and antigen presentation. (A)** Schematic of SLCs activating DCs *in vitro*. **(B-C)** Flow cytometric detection of untreated DCs or DCs treated with SLCs *in vitro*, representative flow cytometry images** (B)** and mean fluorescent intensity (MFI) **(C)** of DCs mature differentiation markers CD40, CD80, CD86, MHCII.** (D)** Schematic of *in vitro* killing assay of CD8^+^ T cells. CD8^+^ T cells isolated from the spleens of C57BL/6 mice were mixed with BMDCs at a 2:1 ratio and incubated with B16F10-GFP cells, with or without the indicated cell amounts of SLCs for 24 h. **(E-F)** Flow cytometry analysis showing the proportion of live B16F10-GFP cells after various treatments. In panel (E), data for each group are presented as mean ± standard deviation (n = 6); ****P < 0.0001 was determined using a two-tailed unpaired t-test. Panel (F) displays representative flow cytometry images.

Tumor cells and cell lysates can be used as tumor antigens, providing a variety of epitopes for vaccine preparation and inducing T cells in the body to produce immune responses. When the body mutates or foreign pathogens invade, DCs are one of the first immune cells to respond. As full-time APCs, mature DCs can initiate the body's innate immunity and promote the activation of antigen-specific T cells to kill tumors. The rough surface presented by SLCs may be more easily recognized by DCs, allowing T cells to more accurately identify and kill tumor cells. Therefore, we further investigated whether SLCs can promote DC maturation and differentiation. As shown in Figure 3A, we co-cultured SLCs with BMDCs isolated from mouse bone marrow *in vitro*, and then used flow cytometry to analyze the expression levels of DC maturation markers CD40, CD80, CD86, and MHC II. As shown in Figure 3B-C, compared with the control group, the expression of DC maturation markers was significantly increased in the SLCs and BMDCs co-culture group, indicating that SLCs promoted the maturation of BMDCs.

To further investigate whether SLCs can be presented to T cells by DCs so that T cells can kill B16F10 melanoma cells, we extracted BMDCs from mouse bone marrow and CD8^+^ T cells from mouse spleen, and then designed a co-culture experiment with B16F10-GFP cells as shown in Figure 3D. After co-culture, the GFP signal is analyzed by flow cytometry, which can reflect the proportion of tumor cell survival. As shown in Figure 3E-F, the addition of SLCs, CD8^+^ T cells, DCs, or any pairwise combination of these components did not have a significant killing effect on B16F10-GFP cells. When SLCs, BMDCs and CD8^+^ T cells were added simultaneously, the B16F10 -GFP signal was significantly reduced in a dose-dependent manner with the amount of SLCs added. The above results indicate that SLCs must be presented to T cells by DCs in order to activate T cells and thereby produce significant killing effects on tumor cells.

### SLCs and immune adjuvants are used to form SLC vaccines, which have stronger antitumor effects

**Figure 4:**
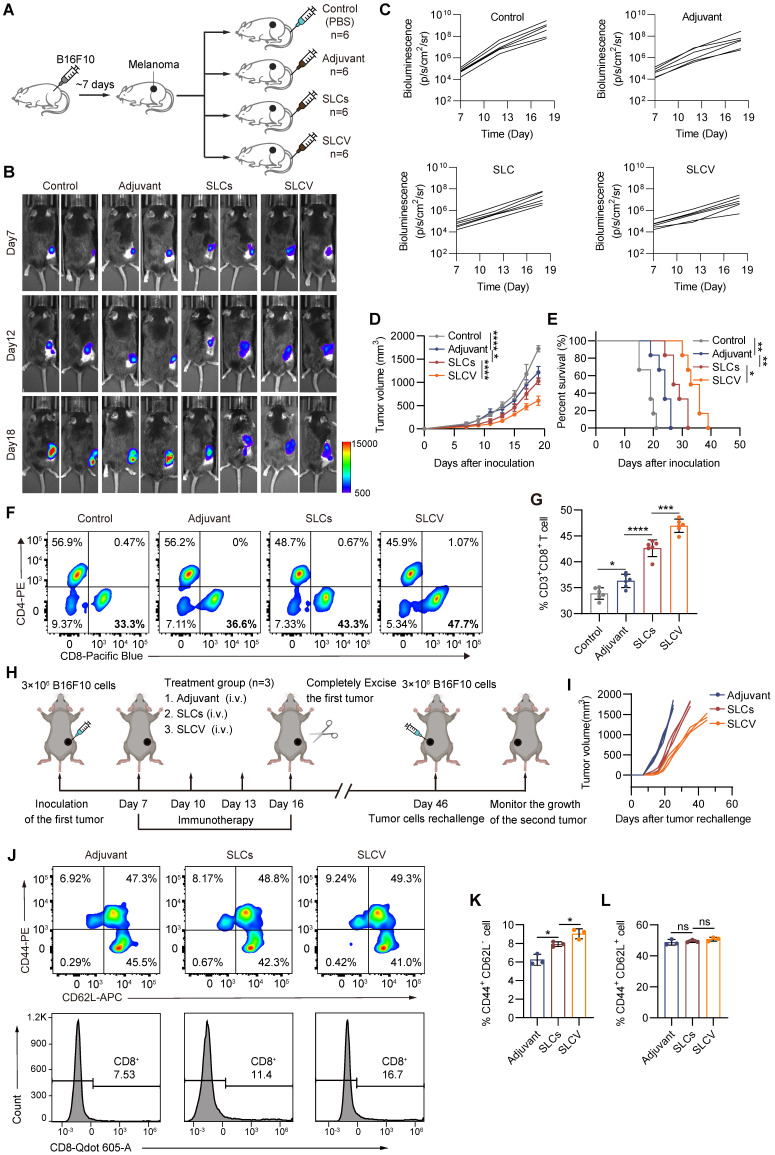
** Enhanced antitumor immunotherapy efficacy of SLC vaccine. (A)** Schematic of model construction and *in vivo* treatment. C57BL/6 mice were subcutaneously inoculated with Tumor cells for 7 days and then intravenously injected (i.v.) with PBS, Adjuvant (MPLA, 20 μg/mouse), SLCs (1 × 10^6^), or SLCV (combined treatment with MPLA and SLCs) every three days, 3 times in total. Tumor tissues were collected on day 19 to analyze the immune response (n = 6). **(B)** Representative bioluminescence images and quantitative bioluminescence **(C)** of mice in different treatment groups (n = 6). **(D)** Tumor volumes were recorded every two days until day 19 (n = 6). **(E)** Kaplan-Meier survival curves of the mice of different treatment groups (n = 6). **(F-G)** Representative flow cytometry data for frequency **(F)** and quantification **(G)** of tumor infiltrating CD8^+^ T cells (n = 6). Data represent analyses of the indicated n mice per group, means ± S.D, and were analyzed by one-way two-sided ANOVA with GraphPad Prism software. *P < 0.05; **P < 0.01; ***P < 0.001; ****P < 0.0001. **(H-K)** Long-term immune memory effects of SLC vaccine treatment.** (H)** Schematic representation of tumor rechallenge. B16F10 cells were injected subcutaneously into the right side of C57BL/6 mice to inoculate the first tumor. When the tumor volume reaches 80-100 mm^3^, the tumors were completely removed after three rounds of treatment with Adjuvant or SLCV. Thirty days after the first tumor was completely excised from the mice, B16F10 cells were again inoculated on the contralateral side to form a second tumor. **(I)** Tumor growth of the rechallenged tumors was recorded. **(J)** Flow cytometric analysis of representative CD8⁺ T cells and memory T cell markers CD62L and CD44 in splenic lymphocytes (gated on CD3^+^ cells) in mice before they were rechallenged to secondary tumors, and quantification of TEM **(K)** and TCM **(L)** in the spleen (n = 3). Data represent means ± S.D, and were analyzed by two-tailed unpaired t tests with GraphPad Prism software. ns, not significant, P > 0.05; *P < 0.05.

Since we have seen that SLCs can stimulate the maturation and differentiation of DCs, thereby promoting T cells to kill tumors, we further explored the possibility of developing SLCs into therapeutic vaccines against tumors. Vaccines typically consist of antigens and adjuvants. SLCs serve as antigens to stimulate immunity, and adjuvants can enhance the immunogenicity of antigens and induce stronger immune responses. Therefore, we combined SLCs with the immune adjuvant monophosphoryl lipid A (MPLA) to form the SLC vaccine (SLCV) and further evaluated the antitumor effect through animal experiments. As shown in Figure 4A, we subcutaneously inoculated B16F10 cells into C57BL/6 mice to evaluate the therapeutic effect of tail vein injection of SLCV on melanoma. The *in vivo* animal imaging results are shown in Figure 4B-C. The tumor volume of the SLCV group was smaller than that of the SLCs group, and the tumor growth rate was also slower (Figure 4D). The survival time of mice in the SLCV group was also significantly longer than that of other groups (Figure 4E). This indicates that SLCV is more effective than pure SLCs in inhibiting tumor growth and prolonging the survival of mice. Compared with the SLCs group, the proportion of infiltrating CD3^+^ CD8^+^ T cells in the tumor tissue treated with SLCV was further increased (Figure 4F-G).

To validate that the vaccine activated tumor-specific immune responses and that such responses were detectable in treated mice, splenocytes from mice subjected to different treatment regimens were analyzed using the B16F10 tumor antigen-specific T cell detection kit. Quantitative analysis revealed a significant increase in tumor antigen-specific T cells in the vaccine-treated group compared to controls (Figure S3G-H). The safety of SLCV is extremely important. Therefore, we performed both acute toxicity (24 h) and long-term toxicity (30 days) studies. For the acute toxicity study, we administered PBS, 1x, 2x, and 10x doses of SLCV to mice and monitored changes in body weight, body temperature, and behavior within 24 h. As shown in Figure S3I-J, none of the groups exhibited significant fluctuations in body weight or temperature, nor did they display abnormal behaviors such as convulsions, coma, lethargy, or increased aggression. We then extended the observation period to 30 days to assess long-term toxicity. The PBS, 1x, and 2x dose groups received SLCV every three days, while the 10x dose group did not receive additional doses after the initial administration. At the end of the experiment, we performed autopsies to examine various organs and conducted HE staining of the liver and kidneys. Throughout the experiment, the mice remained in good condition, with no abnormalities in pupils, respiration, or body temperature. There were no incidents of convulsions, coma, lethargy, increased aggression, or mortality. All mice showed weight gain, with no significant differences between the groups (Figure S3K-L). After 30 days, gross examination of the organs, particularly the liver and kidneys, revealed no significant abnormalities, and HE staining showed no apparent pathological changes in the SLCV-treated groups (Figure S3M-P).

Long-term immune memory is one of the keys to evaluate the antitumor efficacy of vaccines. To investigate whether SLCV treatment can induce long-term immune memory *in vivo* after tumor resection, we established the tumor recurrence model shown in Figure 4H. The mice were rechallenged by inoculating B16F10 cancer cells for the second time on day 46, to evaluate the long-term immune memory effect of SLCV. The results showed that the growth of reinoculated tumors in the SLCV-treated group was significantly inhibited compared with the adjuvant group (Figure 4I). We isolated murine splenocytes on day 46 (corresponding to the second B16F10 tumor inoculation) and quantified the frequency of CD8⁺ T cell subsets, including effector memory T cells, in each group using flow cytometry. The results showed that injection of SLCV significantly increased the proportion of effector memory T cells (CD3^+^ CD8^+^ CD44^+^ CD62L^-^) (Figure 4J-L). The overall results indicate that tail vein injection of SLCV vaccine can induce effector immune memory, which is important for long-term prevention of tumor recurrence.

We further investigated the tumor-initiating capacity in mice after injection with SLC and SLCV. As shown in Figure S4A, pre-immunization with SLC/SLCV followed by subcutaneous transplantation of B16F10 tumors leads to a reduction in both tumor formation rate and tumor-initiating cell frequency. This indicates that SLCV has the potential to prevent tumor formation.

Since there are many similarities between FeCl_3_ and PFA-induced cell death, we further evaluated PFA-fixed cells using the subcutaneous B16F10 melanoma model. The results (Figure S4B-C) showed that MASK cells (SLC) had superior antitumor effects compared to PFA-fixed cells. We conducted an experiment comparing the antigen-presenting capability of live cells (Con), paraformaldehyde-fixed cells (PFA), and SLC to DCs (Figure S4D-E). The results show that after co-culturing the three types of cells with DCs, the proportion of MHC II-positive DCs was significantly higher in the SLC-treated group compared to the live and PFA-fixed cell groups. This indicates that SLC cells are more readily recognized by DCs, facilitating DC maturation and antigen presentation. Furthermore, we added CD8^+^ T cells and GFP-labeled B16F10 cells to the co-culture system. By detecting the B16F10-GFP signal through flow cytometry, we evaluated the differences in T cell-mediated cytotoxicity against tumor cells after DCs were treated with the three types of cells. The results demonstrate that co-culturing SLC cells with DCs and T cells led to a stronger cytotoxic effect on tumor cells compared to live cells and PFA-fixed cells.

Relevant studies have previously reported 13 that liquid nitrogen-treated tumor cells (LNT) can be used as cancer vaccines and promote antitumor immune responses, thereby prolonging the survival time of tumor-bearing mouse models. Therefore, we also compared SLCV with LNTV to investigate whether the SLCV we prepared had more significant antitumor ability. C57BL/6 mice subcutaneously inoculated with B16F10 cells were administered by tail vein injection, as shown in Figure S4F-G, compared with the adjuvant alone group, both the LNTV group and the SLCV group had better therapeutic efficacy, and the effect of tumor growth inhibition in the SLCV group was significantly better than that of LNTV (Figure S4H). SLCV also showed better therapeutic effects in terms of prolonging the survival of tumor-bearing mice (Figure S4I). We then determined the proportion of tumor infiltrating T cells by flow cytometry. As shown in Figure S4J, the proportion of infiltrating CD3^+^ CD8^+^ T cells in the tumor tissue of the SLCV group was significantly increased compared with LNTV. The above results show that SLCV has a more significant therapeutic effect than LNTV in tumor cell vaccines, there is no doubt that this has more positive significance for the research of tumor therapeutic vaccines.

### Magnetic Sculpture-like (MASK) cells enhance vaccine efficacy through their enrichment in tumors

**Figure 5:**
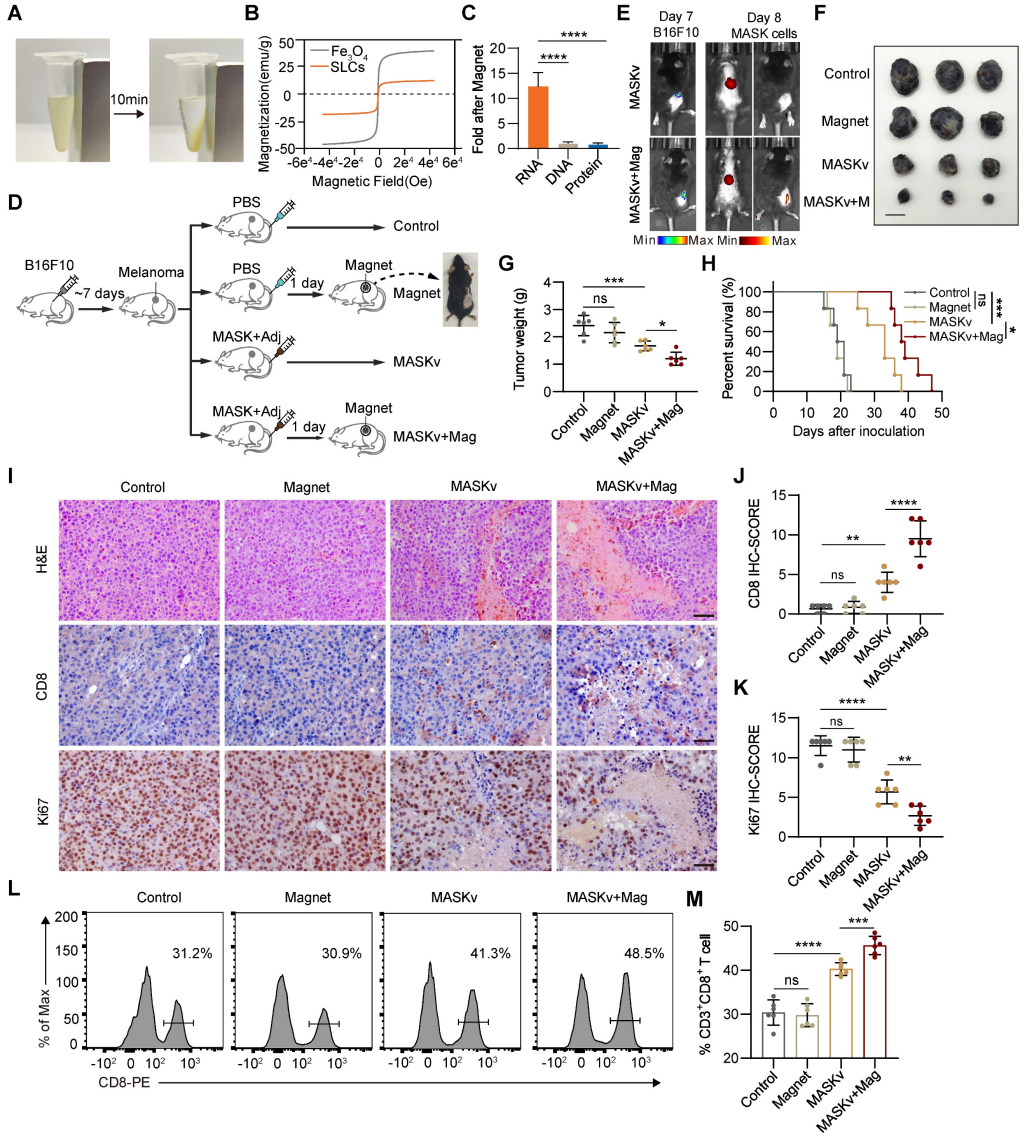
** Magnetic Sculpture-like (MASK) cells enhance vaccine efficacy through their enrichment in tumors. (A)** Digital photographs depicting SLCs before and after 10 min of magnetic field attraction (magnet force = 0.2 T) **(B)** Magnetic hysteresis curves of Fe_3_O_4_ and SLCs at ± 4e^4^ (oe). **(C)** The fold enrichment of DNA, RNA, and protein extracted from MASK cells after magnetic attraction compared to control cells (n = 6). **(D)** Schematic of model construction and magnetic targeted therapy. C57BL/6 mice were subcutaneously inoculated with tumor cells for 7 days, then intravenously (i.v.) injected with PBS or MASKv (combined treatment with MPLA 20 μg/mouse and 1 × 10^6^ MASK cells) every three days for a total of three times, and the tumor tissues were collected on day 19 to analyze the immune response. For MASKv+mag group, an N35 grade NdFeB circular magnet, 8 mm in diameter and 2 mm thick, which we attached to the tumor site with adhesive tape (n = 6). **(E)** Representative bioluminescence image (left) and *in vivo* distribution image of DiR-labeled MASK cells (right) after the first magnetic targeting treatment. **(F)** Image of resected tumor at endpoint and **(G)** tumor weight of resected tumor (n = 6). Scale bar: 1 cm. **(H)** Kaplan--Meier survival curves of the mice of different treatment groups (n = 6). **(I)** HE staining of tumor tissue collected on day 19, immunohistochemical staining of CD8^+^ cells and Ki67. Scale bars, 50 μm. **(J-K)** IHC score of immunohistochemical staining for CD8 **(J)** and Ki67**(K)** (n = 6). **(L-M)** Representative flow cytometry figures **(L)** and quantification **(M)** of tumor infiltrating CD8^+^ T cells of mice. Data represent analyses of the indicated n mice per group, means ± S.D, and were analyzed by one-way two-sided ANOVA with GraphPad Prism software. ns, not significant, P > 0.05; *P < 0.05; **P < 0.01; ***P < 0.001; ****P < 0.0001.

Since SLCs are induced by adding FeCl_3_ solutions to cells and the SLCs suspension turns yellow after centrifugation, it may interact with iron and be endowed with certain iron properties. Magnetism is a property of iron and is relevant for targeted therapy, so we investigated whether SLCs are magnetic. We placed the suspension of SLCs on a magnetic stand. After about 10 min, we were very surprised to find that the SLCs were obviously enriched on one side of the magnet, indicating that SLCs were magnetic (Figure 5A). We then performed a magnetic test on the SLCs, and the hysteresis loop verified that the SLCs were magnetic (Figure 5B). Therefore, we also refer to SLCs as Magnetic Sculpture-like (MASK) cells. Although the magnetic properties of MASK cells of the same mass are weak compared to Fe_3_O_4_, they still have potential for targeted therapy. We preliminarily investigated which biomolecule might contribute to the magnetic properties of MASK cells. DNA, RNA, and protein were extracted from both B16F10 cells and B16F10 MASK cells. After enrichment with a magnetic field of 0.2 T for 10 min, the concentrations were measured. The results showed that the RNA extracted from MASK cells had a higher concentration after magnetic enrichment, which suggests that the magnetic properties of MASK cells might be attributed to RNA (Figure 5C, S5A). This finding was quite unexpected, and the underlying mechanism is difficult to study. It may require further investigation by researchers in the fields of biophysics and chemical biology. Nevertheless, this does not affect our evaluation of whether MASK cells can enhance therapeutic efficacy with the aid of a magnetic field.

Although cancer immunotherapy has made great progress, for solid tumors, since most antigens on the surface of solid tumor cells also exist in normal tissues, and there will be tumor cell heterogeneity within a solid tumor 27, 28. Therefore, when the body's immune function is activated, normal tissues will also be attacked by T cells and cause irreversible damage. A major challenge for immunotherapy of solid tumors is how to activate immunity at targeted tumor sites. Therefore, we investigated whether the magnetism of MASK cells can cooperate with the magnets (Mag) placed at the cancer nest to activate the immunity of the tumor site more efficiently and further improve the effect of tumor immunotherapy. We designed an animal experiment as shown in Figure 5D. On the 7th day after inoculation of B16F10 in mice, melanoma signals could be detected by *in vivo* imaging (Figure 5E). Therefore, we placed magnets at the tumor site one day after inoculation with MASK cell vaccine (MASKv) and used *in vivo* imaging to track MASK cells. We can see that MASK cells are highly enriched at the tumor site (Figure 5E), indicating that MASK cells have sufficient magnetism to be distributed to a specific location under the influence of a magnetic field. After observing mouse survival and tumor volume, we found that the efficacy of MASKv was further enhanced under magnetic “navigation”, the tumor volume was significantly reduced compared with MASKv (Figure 5F-G), and the survival period was significantly prolonged (Figure 5H).

HE staining of the tumor was performed, and the results are shown in Figure 5I. Compared with the control group, the necrotic area in the tumor area of the MASKv group increased, and the tumor necrotic area of the MASKv+Mag group was larger than that of SLCV. CD8 IHC can mark the degree of immune infiltration in the tumor area. The results showed that the immune infiltration of MASKv was significantly higher than that of the control group. The degree of immune infiltration in the MASKv+Mag group was further increased compared with SLCV (Figure 5I-J). The above results show that under magnetic navigation, MASKv can effectively locate the tumor area and increase the degree of immune infiltration in the tumor area. At the same time, the IHC results of the tumor cell proliferation marker Ki67 (Figure 5K) showed that the Ki67 score of tumor cells in the MASKv+Mag group was significantly reduced, indicating that the tumor proliferation ability was weakened after treatment with SLCV+Mag. As shown in Figure 5L-M, compared with the control group, the proportion of infiltrating CD3^+^ CD8^+^ T cells in the tumor tissue of the MASKv+Mag group was significantly increased.

We used the H22-luc liver orthotopic transplant tumor model to observe the impact of a magnetic field on the distribution of MASK signals *in vivo* after injecting MASKv. As shown in Figure S5B, the red signal (MASKv) in the group without a magnet is relatively weak and dispersed throughout the mouse's body, with a slightly stronger signal in the abdominal area. However, after binding a magnet to the mouse's abdomen for one day, the red signal was localized in the liver region and overlapped with the liver cancer area (green). We collected blood samples at different time points within 0 to 24 h after tail vein injection of MASKv to detect the MASK signal. As shown in Figure S5C, the concentration-time curve indicates that, compared to the group without a magnet, the decline in peripheral blood MASK concentration in the magnet group was slower, with an extended half-life. This indicates that MASKv can be directionally enriched in the magnetic field area, increasing the concentration of MASK in that region and slowing its clearance.

In conclusion, our results demonstrate that magnetic MASKv can be enriched into solid tumor nests under the action of external magnetic fields, thereby activating immunity at a targeted location and improving the efficacy of immunotherapy.

### Spatial transcriptomics reveals MASK vaccine has immunotherapy efficacy

**Figure 6:**
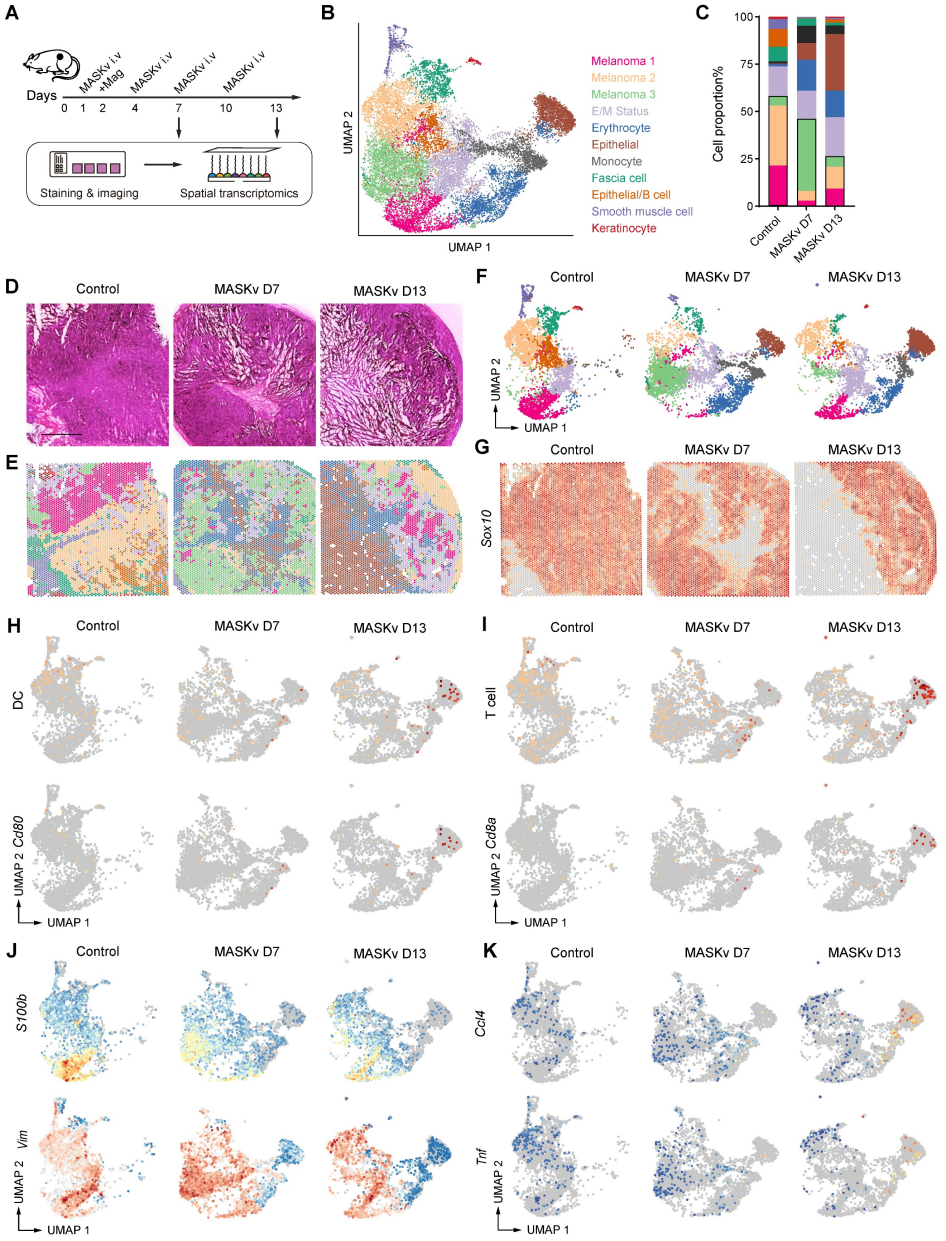
** Spatial transcriptomics of the structural heterogeneity of Tumors and TME in response to magnetic MASK vaccine treatment. (A)** Graphical overview of spatial transcriptomics experimental design. **(B)** Uniform manifold approximation and projection (UMAP) plots of integrated ST spots from the tumor tissues of Control, MASKv D7 and MASKv D13. **(C)** Bar graph showing the proportion of each cell cluster in each sample in the ST dataset. **(D)** H&E staining of three tumor tissue samples, and **(E)** unbiased cluster analysis of ST spots. **(F)** UMAP plots of ST spots for three tumor tissue samples, respectively. **(G)** The spatial feature maps illustrate the spatial expression of Sox10 in each tumor tissue. **(H-I)** The spatial expression maps of dendritic cell markers (CD40, CD80, CD86) and CD80 in tumor tissues **(H)**, and T cell markers (CD3E, CD3G, CD8A, IKZF2, THY1) as well as CD8A in tumor tissues **(I)**. **(J)** The spatial expression maps of Vimentin and S100B in tumor tissues of different treatment groups. **(K)** The spatial expression maps of CCL4 and TNF in tumor tissues of different treatment groups.

To explore the pharmacological effects on tumors and TME structural heterogeneity under MASKv treatment, we performed spatial transcriptomics (ST) with B16F10 xenograft receiving MASKv and magnet treatment (Figure 6A). After quality control, based on unbiased clustering and spot features, spots were classified as 3 clusters of melanoma cells, E/M Status with high expression of Espn, Mpp2, Ndrg1 and Fosl2, erythrocytes with high expression of Hba-a2, Hbb-bs and Hbb-bt, epithelial cells with high expression of Wee1, Nr2f6 and Pnrc1, monocytes/T cells with high expression of Il1b, G0s2, Cxcl3, Cd14 and Ccl4, fascia cells with high expression of Col3a1 or keratinocyte with high expression of Krt5 and Krt14 (Figure 6B). We analyzed the proportion of each cell subpopulation and found that the proportion of melanoma cells decreased after MASKv treatment, especially when it dropped to 25% after 13 days of MASKv treatment, indicating that MASKv has a better killing effect on melanoma (Figure 6C). Figure 6D shows the HE staining of mouse melanoma tissues in the control group and after MASKv treatment for 7 days and 13 days. Figure 6E shows the results of cell type annotations corresponding to the spatial position. Figure 6F shows the cell grouping characteristics of the three samples.

The reported spatial expression of the melanoma signature gene *Sox10* (Figure 6G) shows that Sox10^+^ cells were significantly reduced after MASKv treatment, further supporting that MASKv can have a therapeutic effect on tumors. The total spatial expression of DC markers (CD40, CD80, CD86) 29 and the spatial expression of CD80 (Figure 6H) showed that after MASKv treatment, CD80^+^ cells increased significantly, and DC maturation marker expression increased, and DC maturation marker-positive cells increased significantly, indicating that MASKv increased the maturation of DCs in tumors and promoted their antigen presentation. The overall spatial expression of T cell markers 30 and the spatial expression of CD8 (Figure 6I) showed that after MASKv treatment, CD8^+^ cells increased significantly, the expression of T cell markers increased, and the number of positive cells increased significantly. This indicates that MASKv promotes the number of CD8^+^ T cells, enhances the function of T cells, and promotes the killing effect of T cells on tumors. Further analysis of the spatial transcriptome data showed that the expression of marker genes *S100b* and vimentin related to melanoma malignant progression and metastasis 31, 32 were downregulated after MASKv treatment (Figure 6J), while inflammatory factor-related genes such as *Ccl4* and* Tnf* 33 were upregulated after MASKv treatment (Figure 6K), indicating that MASKv activated tumor immunity and inhibited the malignant evolution of tumors. We further analyzed the impact of SLCV on various immune cells using mMCP-counter. As shown in Figure S6, in addition to DC cells and T cells, the scores for memory B cells (associated with long-term immunity), NK cells (associated with non-specific immunity), eosinophils, neutrophils, and mast cells (all related to allergic reactions) increased after SLCV treatment. This suggests that SLCV may activate these immune cells. Conversely, the score for regulatory T cells decreased after SLCV treatment, indicating that SLCV may enhance tumor immunity by inhibiting regulatory T cells. Unexpectedly, the scores for macrophages/monocytes decreased after SLCV treatment, implying that SLCV might have an inhibitory effect on these cells. These findings demonstrate that SLCV has complex direct or indirect effects on various immune cells. We performed live-cell imaging to observe whether macrophages could swallow MASK cells. Video S2 shows that although RAW264.7 macrophages exhibit behavior around MASK cells, the number of MASK cells does not significantly decrease after 24 h, and the morphology of MASK cells remains relatively intact. This suggests that MASK cells are not easily phagocytosed by macrophages.

### Magnetically targeted MASK vaccine combined with PD-1 inhibitor has stronger antitumor effect

While we have demonstrated that the MASK tumor cell vaccine can effectively stimulate tumor infiltrating T cells, T cell activity is reduced due to the influence of the immunosuppressive tumor microenvironment. In various murine tumor models, tumor specific CD8^+^T cells exhibit characteristics of T cell exhaustion and dysfunction 34, thus weakening the therapeutic effect of tumor cell vaccines. Clinically, checkpoint blockade therapy with PD-1 and PD-L1 antibodies has been shown to significantly improve T cell depletion caused by the immunosuppressive tumor microenvironment. Tumor cell vaccines can be combined with checkpoint blockade therapy to enhance immunotherapy effect. Therefore, we combined MASKv with anti-PD-1 antibody to verify its antitumor efficacy.

**Figure 7:**
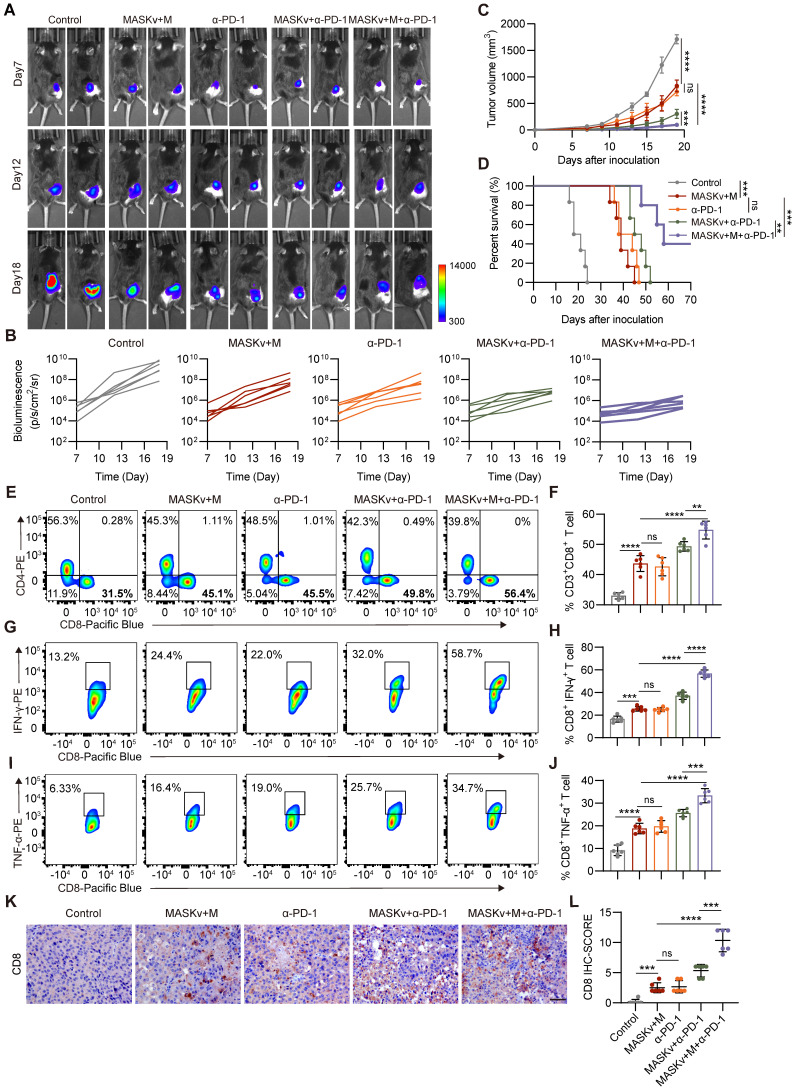
** MASK vaccine combined with PD-1 inhibitor enhances the efficacy of ICB immunotherapy. (A)** Representative bioluminescence images and quantitative bioluminescence **(B)** of mice in different treatment groups. **(C)** Tumor volumes were recorded every two days until day 19. **(D)** Kaplan-Meier survival curves of the mice of different treatment groups. **(E-F)** Representative flow cytometry data **(E)** and quantification **(F)** of tumor infiltrating CD8^+^ T cells. **(G-H)** Representative flow cytometry data **(G)** and quantification **(H)** of IFN-γ^+^ CD8^+^ T cells. **(I-J)** Representative flow cytometry data **(I)** and quantification **(J)** of TNF-α^+^ CD8^+^ T cells. **(K)** Immunohistochemical staining of CD8^+^ cells. Scale bar, 50 μm, and IHC score **(L)** of CD8 (n=6). Data represent analyses of the indicated 6 mice per group, means ± S.D, and were analyzed by one-way two-sided ANOVA with GraphPad Prism software. ns, not significant, P > 0.05; **P < 0.01; ***P < 0.001; ****P < 0.0001.

We found that a combination of intravenous MASKv administration and intraperitoneal anti-PD-1 antibody achieved striking results. As shown in Figure 7A-C, MASKv combined with anti-PD-1 almost prevented tumor growth and greatly prolonged the survival period of mice. On the 60th day after tumor inoculation, half of the mice were still alive (Figure 7D). Tumor samples were collected from tumor-bearing mice and analyzed by flow cytometry (Figure 7E-F). The results showed that tumor infiltrating CD8^+^ T cells were significantly increased in the combination treatment group.

Cytotoxic T lymphocytes (CTL) can directly kill tumor cells by releasing perforin or granzymes and are the key to immunotherapy. Therefore, we investigated the CTL induced in the combination treatment group. IFN-γ is a typical marker of CTL cytotoxic activity. We measured the content of IFN-γ^+^ CD8^+^ T cells in each group by flow cytometry and found that the number of IFN-γ^+^ CD8^+^ T cells was significantly increased in the combination treatment group (Figure 7G-H), indicating that the combined treatment group effectively promoted the generation of CTL. Similarly, TNF-α^+^ CD8^+^ T cells (Figure 7I-J) were also significantly increased in the combination treatment group. At the same time, the IHC results showed that the staining intensity and the percentage of CD8^+^ T cells were significantly higher in the combination treatment group (Figure 7K-L), consistent with the flow cytometry data.

The efficacy of cancer immunotherapy will vary widely in different tumor environments. In particular, tumor cell models overexpressing OVA have higher immunogenicity 35, so ICB immunotherapy may activate a more potent immune response. There are also related studies reporting that MC38 tumors are extremely sensitive to PD-1 36. Therefore, we evaluated the therapeutic efficacy of MASKv combined with anti-PD-1 antibody in B16 OVA and MC38 tumor models. We found that in the mouse subcutaneous tumor model, the combined treatment group had a very significant effect in the B16 OVA and MC38 tumor models, with tumor growth strongly inhibited and survival rate significantly improved (Figure S7A-C). At the same time, we obtained the same results in the mouse subcutaneous tumor recurrence model (Figure S7D-F). These results demonstrate that the treatment regimen of MASKv combined with anti-PD-1 antibody can have very excellent efficacy in various tumor models and has great prospects for clinical application.

## Discussion

In this study, we discovered a new way of cell death—sculpture-like death. After cells are treated with high concentration of FeCl_3_, the cells become magnetic and have a mask-like shape, so they are called Magnetic Sculpture-like (MASK) cells. Sculpture-like death differs morphologically from cell fixation induced by PFA, with cells exhibiting a rougher surface. All the cells we tested can undergo sculpture-like death, and this type of death can also be observed when high concentrations of FeCl_3_ are directly injected into living tissues, indicating that the induction of MASK cells by high concentrations of FeCl_3_ is efficient and universal. Sculpture-like death is not a programmed cell death (such as apoptosis, autophagy, ferroptosis, etc.) because the process of cell sculpture-like death takes less than one min. Such a short time is more consistent with the characteristics of chemical changes.

A large number of studies have shown that tumor cells can be used as therapeutic vaccines for tumors 16, 37, 38. Compared with other whole tumor cell vaccines, MASK cells are unique in that they are treated with FeCl_3_, so MASK cells should contain a certain amount of iron. In addition to providing magnetism, iron is an important component of the blood. Studies have shown that iron can promote the formation of neutrophils and the production of inflammatory factors 39, 40, so MASK cells show a strong activating effect on the immune system. In addition, considering that iron overload is a condition in which excess iron is deposited in various organs 41, which may lead to the formation of MASK cells, the presence of MASK cells in related diseases remains to be investigated.

The future clinical application of MASK cells is the preparation of personalized autologous anonymous antigen vaccines. Specifically, the patient's tumor tissue is obtained by surgery or biopsy and treated *in vitro* with high concentrations of FeCl_3_ to obtain MASK cells, which are combined with immune adjuvants to form a MASK cell vaccine, which is then reinfused into the patient's body by intravenous injection. Because the MASK cell antigen is derived from the patient's own tumor, it is more specific. The unique magnetic properties of MASK cells also allow them to be enriched in the tumor area through magnetic field navigation. The safety and efficacy of this therapy were evaluated in this study in a mouse model. The combination of MASKv and PD-1 antibodies under magnetic navigation can further enhance the antitumor effect and significantly prolong survival.

MASKv has four advantages. 1.Easy to prepare: Just add FeCl_3_ solution to the cells at room temperature, and it can be obtained in less than 1 min without repeated operations; 2. Cost-effective, the price of FeCl_3_ and simple operation save a lot of cost; 3. Effective: The rough shape of MASK cells and the iron they contain can further enhance the immune response. In addition, MASK cells retain their complete shape and are structurally more rigid under the action of iron, without being brittle caused by repeated freezing and thawing, and adhesion problems. Our data show that MASKv has a better effect on B16F10 subcutaneously transplanted tumors than previously reported liquid nitrogen treated B16F10 cells; 4. Navigable: MASKv can cooperate with magnetic fields to achieve directional enrichment and treatment of solid tumors.

In accordance with conventional wisdom, elevated concentrations of FeCl_3_ typically induce protein salting out. Thus, we initially hypothesized that the magnetism observed in MASK cells stemmed from proteins. However, our findings were remarkably unexpected: the magnetism exhibited by MASK cells did not emanate from proteins or DNA, but rather from RNA. The chemical structure of the pentoses in DNA and RNA is very similar, but the DNA of MASK cells has almost no magnetic effect, whereas RNA has a magnetic effect. This selectivity surprised us. This discovery may have important implications for future RNA research and magnetic biology research. RNA is widely distributed in the nucleus and cytoplasm of cells. In recent years, it has also been found to be distributed on the cell surface 42, 43, so that high-concentration FeCl_3_ treatment enables the whole cell to exhibit a strong and homogeneous magnetic effect, which is an important basis for magnetic navigation, and may also lay the foundation for the future realisation of whole-cell magnetisation and even magnetisation of tissues and organs.

Frankly, this study serendipitously discovered that high concentrations of FeCl_3_ cause statue-like cell death. However, the molecular mechanisms underlying this process remain elusive, as does the cause of the observed magnetism, particularly regarding how RNA generates magnetism. Further investigation is warranted to elucidate these phenomena. In addition, although this study shows that high concentrations of FeCl_3_ can induce sculpture-like death of various types of cells, the effect of MASKv was studied using mouse B16F10 melanoma as an example, and the efficacy on other types of malignant tumors requires further study.

## Supplementary Material

Supplementary figures.

Video S1. Live cell imaging of PLC-PRF-5 cells forming SLC, related to Figure 1.

Video S2. Live cell imaging of SLC co-cultured with RAW264.7 macrophages, related to Figure S6.
