# Supplementary material for: Magnetic sculpture-like tumor cell vaccines enable targeted in situ immune activation and potent antitumor effects
Source: Theranostics. 2025 Apr 13;15(11):5358–80. doi: 10.7150/thno.107162 (PMC12036876; doi:10.7150/thno.107162)
Supplement: Supplementary file 1 — Supplementary figures. [file thnov15p5358s1.pdf]

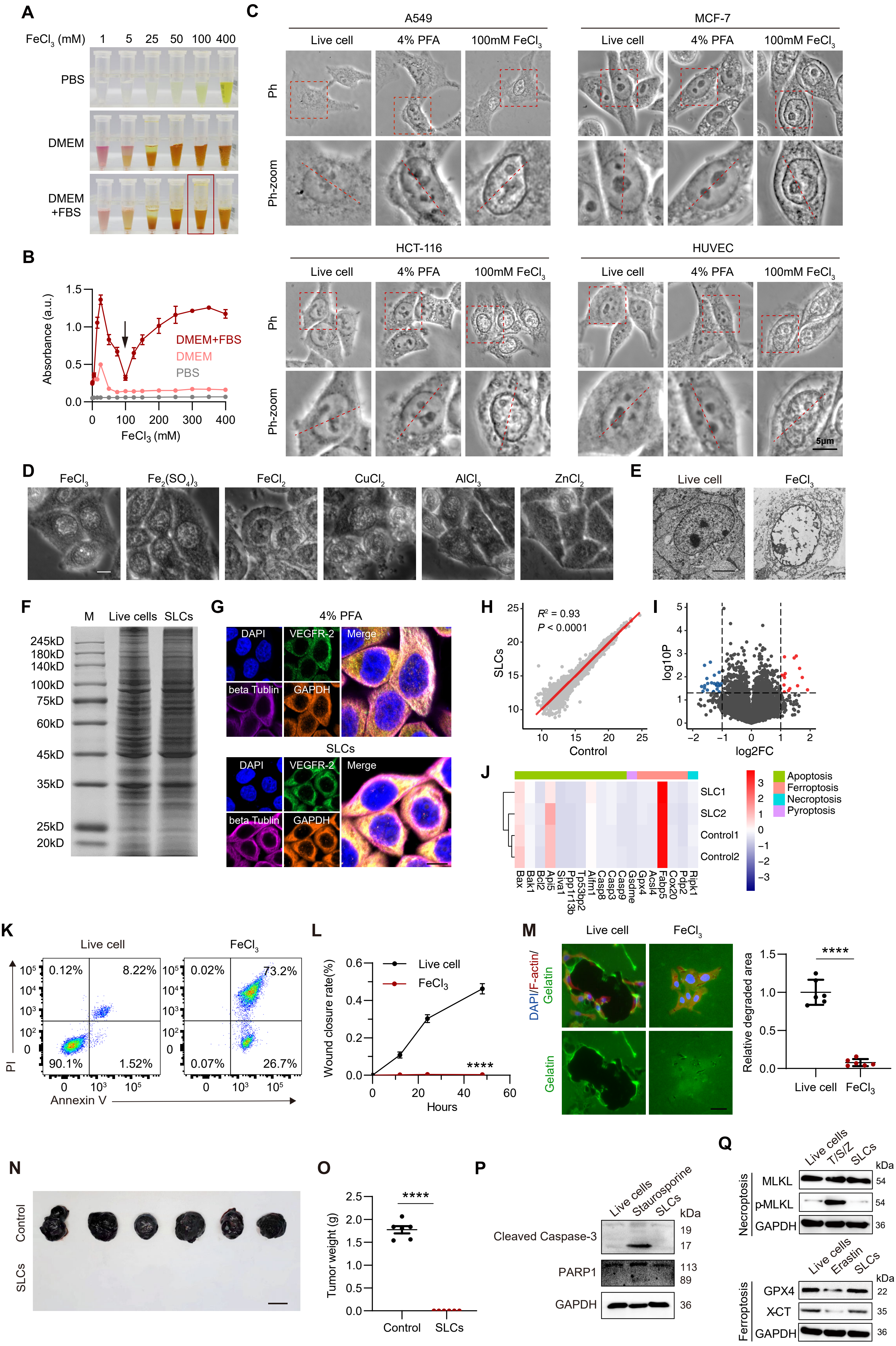

A
FeCl3 (mM) 1 5 25 50 100 400
PBS
DMEM
DMEM +FBS
B
Absorbance (a.u.)
DMEM+FBS
DMEM
PBS
FeCl3 (mM)
C
A549
MCF-7
HCT-116
HUVEC
Live cell
4% PFA
100mM FeCl3
Ph
Ph-zoom
5μm
D
FeCl3
Fe2(SO4)3
FeCl2
CuCl2
AlCl3
ZnCl2
E
Live cell
FeCl3
F
M Live cells SLCs
245kD
180kD
140kD
100kD
75kD
60kD
45kD
35kD
25kD
20kD
G
4% PFA
SLCs
DAPI
VEGFR-2
Merge
beta Tublin
GAPDH
H
R² = 0.93
P < 0.0001
SLCs
Control
I
log10P
log2FC
J
SLC1
SLC2
Control1
Control2
Apoptosis
Ferroptosis
Necroptosis
Pyroptosis
Bax Bak1 Bcl2 Api5 Siva1 Ppp1r13b Tp53bp2 Aifm1 Casp8 Casp3 Casp9 Gsdme Gpx4 Acsl4 Fabp5 Cox20 Pdp2 Ripk1
K
Live cell
FeCl3
0.12% 8.22% 90.1% 1.52%
0.02% 73.2% 0.07% 26.7%
PI
Annexin V
L
Wound closure rate(%)
Live cell
FeCl3
****
Hours
M
Live cell
FeCl3
DAPI/F-actin/Gelatin
Gelatin
Relative degraded area
****
Live cell FeCl3
N
Control
SLCs
O
Tumor weight (g)
****
Control SLCs
P
Live cells Staurosporine SLCs
kDa
Cleaved Caspase-3 19 17
PARP1 113 89
GAPDH 36
Q
Live cells T/S/Z SLCs
kDa
Necroptosis
MLKL 54
p-MLKL 54
GAPDH 36
Live cells Erastin SLCs
kDa
Ferroptosis
GPX4 22
X-CT 35
GAPDH 36

Figure S1. SLCs have no cell viability and tumorigenic ability, related to Figure 1.

(A) Representative images of different concentrations of $FeCl_3$ in PBS, DMEM and DMEM+PBS solutions, and **(B)** statistics of absorbance intensities measured at λ=561 nm (n=3). **(C)**Representative brightfield (top) and phase contrast (middle) microscopy images of A549, MCF-7, HCT-116 and HUVEC cells treated with PBS, 4% paraformaldehyde, or 100 mM $FeCl_3$. The red dashed box represents a magnified view of the phase contrast microscopy image. Scale bar, 5 μm. **(D)** Representative phase contrast microscopy images of PLC-PRF-5 cells treated with 100 mM metal salts including $FeCl_3$, $Fe_2(SO_4)_3$, $FeCl_2$, $CuCl_2$, $AlCl_3$, $ZnCl_2$. **(E)** TEM imaging of ultrathin sections of B16F10 normal and $FeCl_3$-treated cells. Scale bar, 5 μm. **(F)** Coomassie brilliant blue staining of total protein content of PLC-PRF-5 living cells and cells treated with $FeCl_3$, and **(G)** DAPI staining and immunofluorescence staining of β-Tubulin, GAPDH and VEGFR-2. Scale bar,10 μm. **(H)** Scatter plot of protein expression correlation between B16F10 normal and $FeCl_3$-treated cells. **(I)** Volcano plot displaying protein expression changes between B16F10 normal and $FeCl_3$-treated cells. **(J)** Heatmap and hierarchical clustering analysis of programmed cell death biomarkers in proteomics. **(K)** Annexin V-PI flow cytometric analysis of PLC-PRF-5 live cells and cells treated with $FeCl_3$. **(L)** Quantification of the wound healing assay in PLC-PRF-5 live cells or cells treated with $FeCl_3$ for 0, 12, 24, and 48 hours. **(M)** Fluorescent gelatin degradation and phalloidin/DAPI staining of PLC-PRF-5 live cells or cells treated with $FeCl_3$. Quantification of relative fluorescent gelatin degradation is shown in the right panel. Scale bar, 30 μm. **(N-O)** Representative tumor image, scale bar, 5 μm. Tumor weight **(O)** of subcutaneous B16F10 implants in mice as in Fig S1N. **(P)** Western blotting was performed to detect apoptotic markers (Cleaved Caspase-3, PARP1). The apoptosis inducer Staurosporine treated cells were detected as a positive control. **(Q)** Western blotting was performed to detect necroptosis marker (pMLKL), and ferroptosis markers (GPX4 and xCT). The middle lane represents the positive controls for each death pathway: TNF-α (20 ng/ml), 100 nM Sm-164, and 20 μM Z-VAD-FMK induce necroptosis. Erastin (5 μM) induces ferroptosis. Data represent analyses of the indicated n mice per group, means ± S.D, and were analyzed by two-tailed unpaired t tests with GraphPad Prism software. ****$P < 0.0001$.

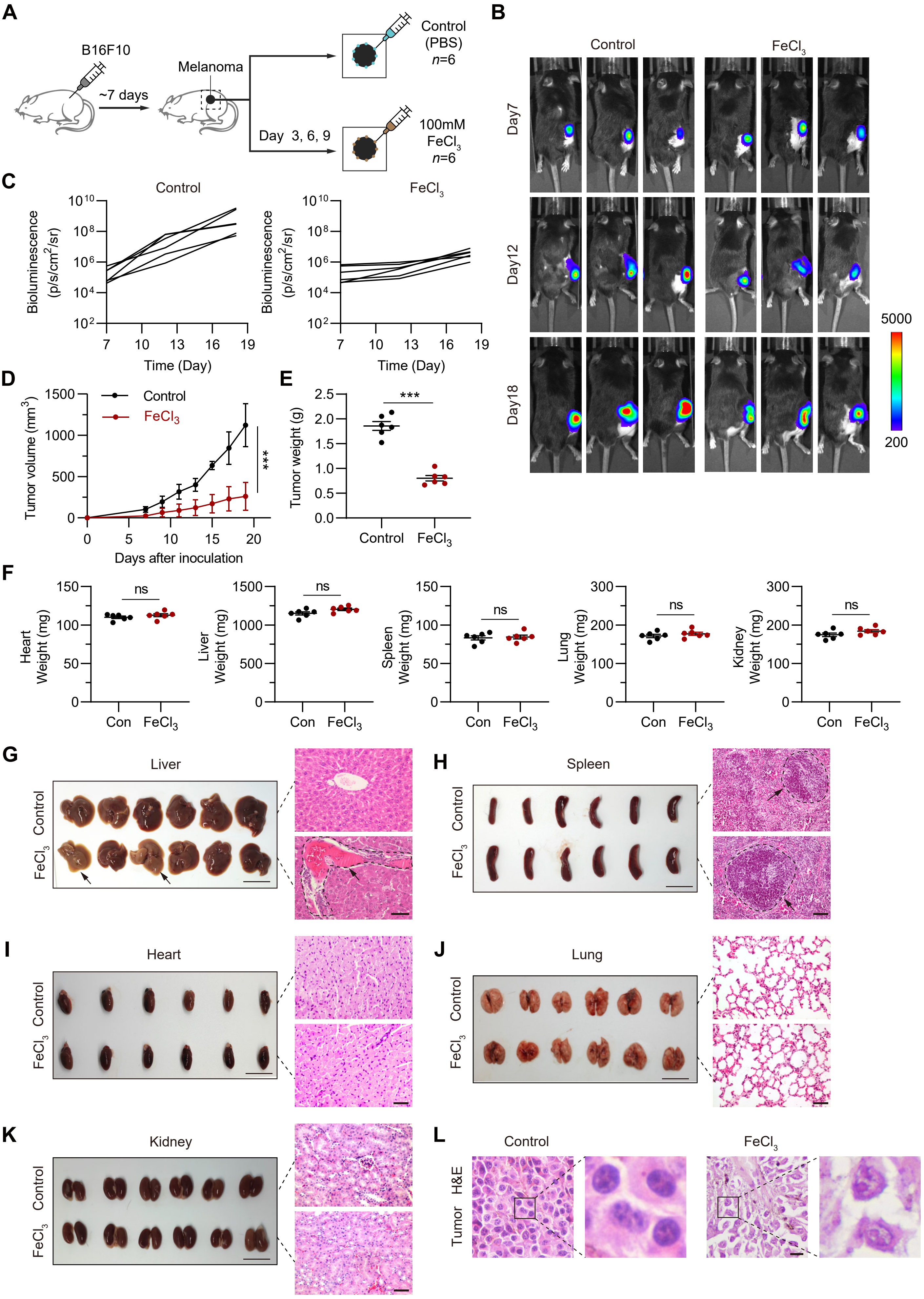
A
B16F10
~7 days
Melanoma
Control (PBS) n=6
Day 3, 6, 9
100mM FeCl3 n=6
B
Control
FeCl3
Day7
Day12
Day18
5000
200
C
Control
FeCl3
Bioluminescence (p/s/cm2/sr)
Time (Day)
D
Control
FeCl3
Tumor volume (mm3)
Days after inoculation
***
E
Tumor weight (g)
***
Control
FeCl3
F
Heart Weight (mg)
Liver Weight (mg)
Spleen Weight (mg)
Lung Weight (mg)
Kidney Weight (mg)
ns
Con
FeCl3
G
Liver
H
Spleen
I
Heart
J
Lung
K
Kidney
L
Control
FeCl3
Tumor H&E

Figure S2. Intratumoral injection of high concentration $FeCl_3$ can limit tumor growth, but needs to be improved, related to Figure 2.

(A) Schematic showing model construction and in situ tumor injection of $FeCl_3$. C57BL/6 mice received subcutaneous injections of B16F10-luciferase cells on day 0. After seven days of tumor formation, 100 mM $FeCl_3$ was injected in situ at multiple points around the tumor every 3 days for a total of 3 injections, and intratumoral injection of PBS served as a control group. The mice were sacrificed on day 19, and the tumor tissues and organs of the mice were collected. **(B-C)** Representative bioluminescence images **(B)** and quantitative bioluminescence **(C)** of mice in different treatment groups (n=6). **(D)** Tumor volumes were recorded every two days until day 19 (n = 6). **(E)** Tumor weight was measured on day 19 (n = 6). **(F)** Hearts, livers, spleens, lungs and kidneys were removed and organ weights were recorded on day 19 (n = 6). **(G-K)** Representative images of livers, spleens, hearts, Lungs and Kidneys (Scale bar, 1 cm) collected on day 19 and HE staining of sections (Scale bar, 100 μm). **(L)** HE staining of tumor tissue. Scale bar, 50 μm. Data represent analyses of the indicated n mice per group, means ± S.D, and were analyzed by two-tailed unpaired t tests with GraphPad Prism software. ns, not significant, $P > 0.05$; *** $P < 0.001$.

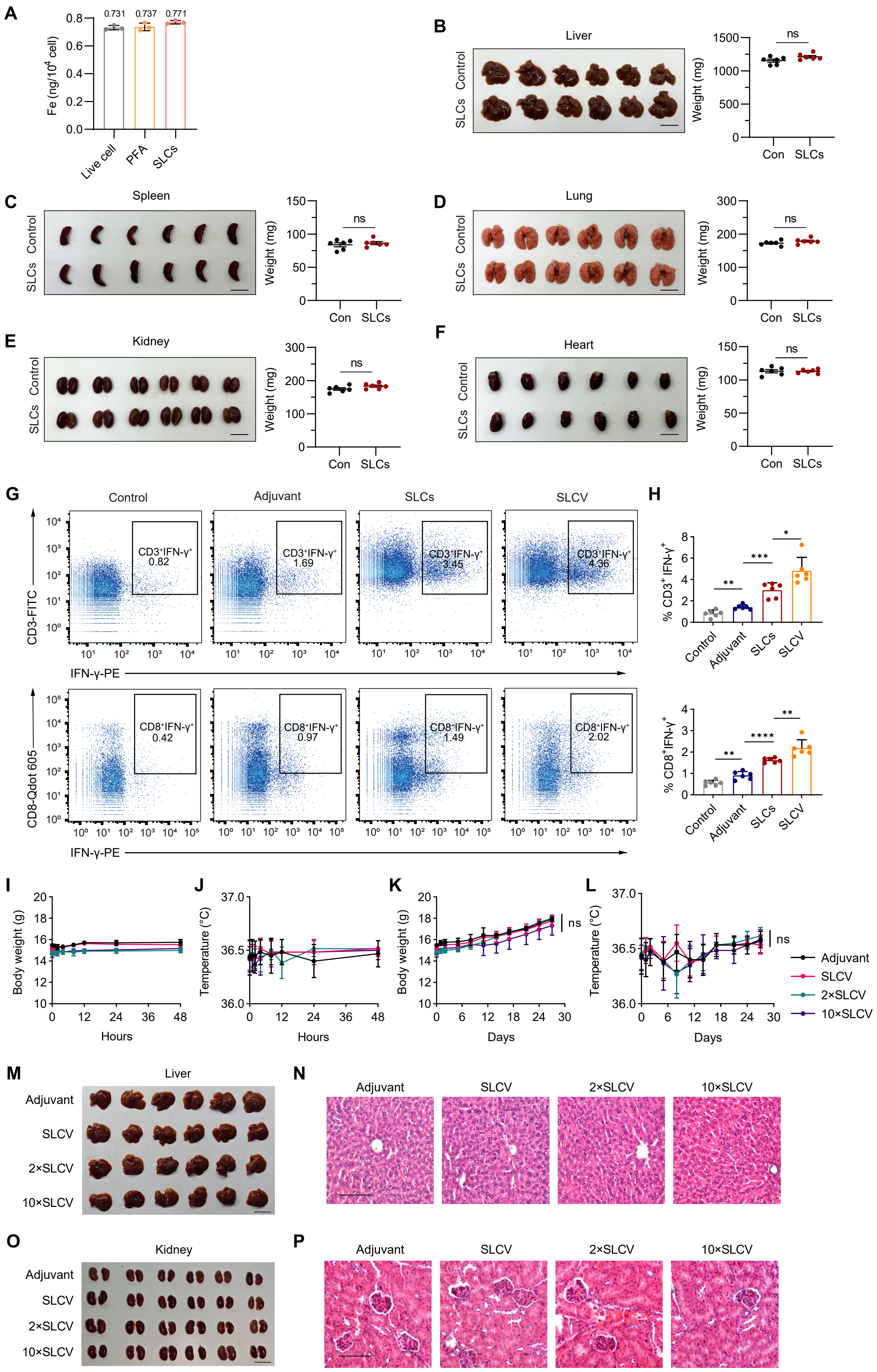

A
0.731
0.737
0.771
Fe (ng/10^4 cell)
Live cell
PFA
SLCs
B
Liver
Control
SLCs
Weight (mg)
ns
Con
SLCs
C
Spleen
D
Lung
E
Kidney
F
Heart
G
Control
Adjuvant
SLCs
SLCV
CD3-FITC
IFN-γ-PE
CD3+IFN-γ+
0.82
1.69
3.45
4.36
CD8-Qdot 605
CD8+IFN-γ+
0.42
0.97
1.49
2.02
H
% CD3+ IFN-γ+
% CD8+IFN-γ+
I
Body weight (g)
Hours
J
Temperature (°C)
K
Days
L
Adjuvant
SLCV
2×SLCV
10×SLCV
M
Liver
N
O
Kidney
P

Figure S3. The treatment with SLCs or SLCV had no detectable toxicity in mice, related to Figures 2 and 4.

(A) Measurement of iron content in B16F10 live cells, B16F10 cells fixed with paraformaldehyde (PFA), and SLC derived from B16F10 cells treated with ferric chloride. **(B-F)** On day 19, Livers **(B)**, Spleens **(C)**, Lungs **(D),** Kidneys **(E)** and Hearts **(F)** were removed (Scale bar, 1 cm) and organ weights were recorded (n=6). Data represent analyses of the indicated n mice per group, means ± S.D, and were analyzed by two-tailed unpaired t tests with GraphPad Prism software. ns, not significant, $P > 0.05$. **(G-H)** Flow cytometric quantification of tumor antigen-specific T cell subsets in different treatment group. **(G)** Representative flow cytometry plots of $CD3^{+}IFN\text{-}\gamma^{+}$ and $CD8^{+}IFN\text{-}\gamma^{+}$ T cells in Control, Adjuvant, SLCs, and SLCV groups. Numerical values indicate the percentage of positive cells within respective gates. **(H)** Bar graphs summarizing the frequencies of $CD3^{+}IFN\text{-}\gamma^{+}$ and $CD8^{+}IFN\text{-}\gamma^{+}$ T cell subsets. Data are presented as mean±SEM; significance was determined by two-tailed unpaired t tests with GraphPad Prism software. ****$P < 0.0001$. **(I-J)** Acute toxicity (24h) experiments for SLCV. We administered PBS, 1x, 2x, and 10x doses of SLCV to mice and monitored changes in body weight **(I)**, body temperature **(J)**, and behavior within 24 hours. **(K-L)** Long-term toxicity (30d) experiments for SLCV. The PBS, 1x, and 2x dose groups received SLCV every three days, while the 10x dose group did not receive additional doses after the initial administration. Changes in body weight **(K)**, body temperature **(L)**, and behavior were monitored. **(M-P)** After 30 days, the liver **(M)** and kidneys **(O)**, revealed no significant abnormalities (Scale bar, 1 cm), and HE staining (Scale bar: 50 μm) showed no apparent pathological changes in liver **(N)** and kidneys **(P)** the SLCV-treated groups.

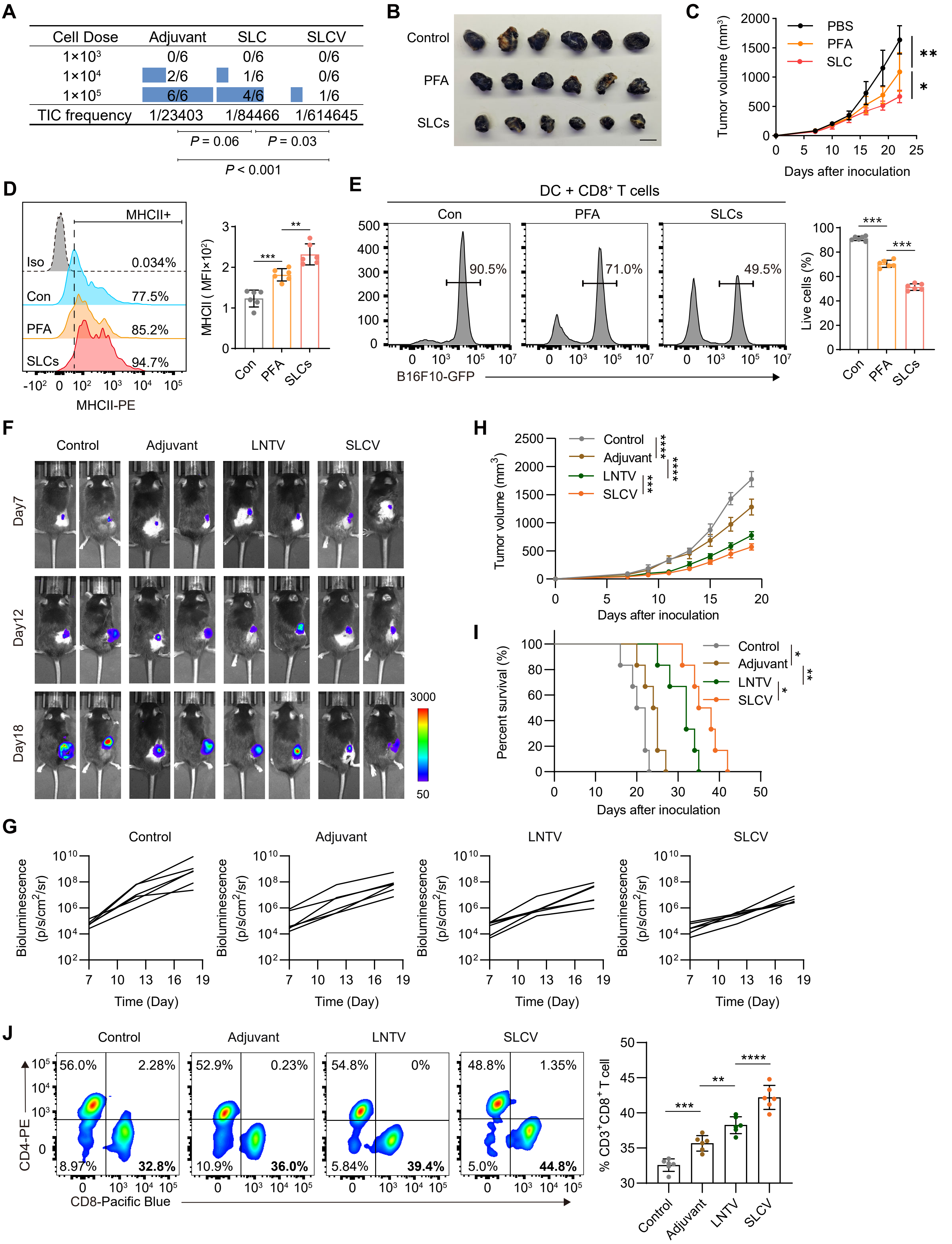
A
Cell Dose
Adjuvant
SLC
SLCV
1×10³
0/6
0/6
0/6
1×10⁴
2/6
1/6
0/6
1×10⁵
6/6
4/6
1/6
TIC frequency
1/23403
1/84466
1/614645
P = 0.06
P = 0.03
P < 0.001
B
Control
PFA
SLCs
C
Tumor volume (mm³)
Days after inoculation
PBS
PFA
SLC
D
MHCII+
Iso
0.034%
Con
77.5%
PFA
85.2%
SLCs
94.7%
MHCII-PE
MHCII (MFI×10²)
Con
PFA
SLCs
E
DC + CD8⁺ T cells
Con
PFA
SLCs
90.5%
71.0%
49.5%
B16F10-GFP
Live cells (%)
F
Control
Adjuvant
LNTV
SLCV
Day7
Day12
Day18
3000
50
H
Control
Adjuvant
LNTV
SLCV
Tumor volume (mm³)
Days after inoculation
I
Percent survival (%)
Days after inoculation
G
Control
Adjuvant
LNTV
SLCV
Bioluminescence (p/s/cm²/sr)
Time (Day)
J
Control
Adjuvant
LNTV
SLCV
56.0%
2.28%
8.97%
32.8%
52.9%
0.23%
10.9%
36.0%
54.8%
0%
5.84%
39.4%
48.8%
1.35%
5.0%
44.8%
CD4-PE
CD8-Pacific Blue
% CD3⁺CD8⁺ T cell
Control
Adjuvant
LNTV
SLCV

Figure S4. Immunotherapy for SLC vaccine is more effective compared to PFA treated cell and LNT vaccine, related to Figure 4.

(A) The tumor-initiating capacity of the cells was assessed using extreme limiting dilution analysis (ELDA). The frequency of B16F0 tumor-initiating cells (TIC) was calculated for the adjuvant, SLC, and SLCV treatment groups. Biological replicates, n=6. The Chi-square test was used to determine the P value. **(B)** Tumor image in different treatment groups. Scale bar: 1 cm. **(C)** Tumor volumes were recorded every three days until day 22 (n=6). **(D)** Flow cytometric detection of untreated DCs or DCs treated with SLCs in vitro, representative flow cytometry images (left) and mean fluorescent intensity (MFI) (right) of DCs mature differentiation marker MHCII. **(E)** $CD8^+$ T cells isolated from the spleens of C57BL/6 mice were mixed with BMDCs at a 2:1 ratio and incubated with B16F10-GFP cells, with indicated cells for 24 h. B16F10-GFP cell viability was analyzed by flow cytometry. **(F-G)** Representative bioluminescence images **(F)** and quantitative bioluminescence **(G)** of mice after treatment with PBS, adjuvant (MPLA, 20 ug/mouse), LNTV (combined treatment with MPLA and 1×10^6 LNT cells) or SLCV (combined treatment with MPLA and 1×10^6 SLCs) (n=6). The treatment process is the same as Fig. 5A. **(H)** Tumor volumes were recorded every two days until day 19 (n = 6). **(I)** Kaplan–Meier survival curves of the mice of different treatment groups (n=6). **(J)** Representative flow cytometry data for frequency and quantification of tumor infiltrating $CD8^+$ T cells (n=6). Data represent analyses of the indicated n mice per group, means ± S.D, and were analyzed by one-way two-sided ANOVA with GraphPad Prism software. *P < 0.05; **P < 0.01; ***P < 0.001; ****P < 0.0001.

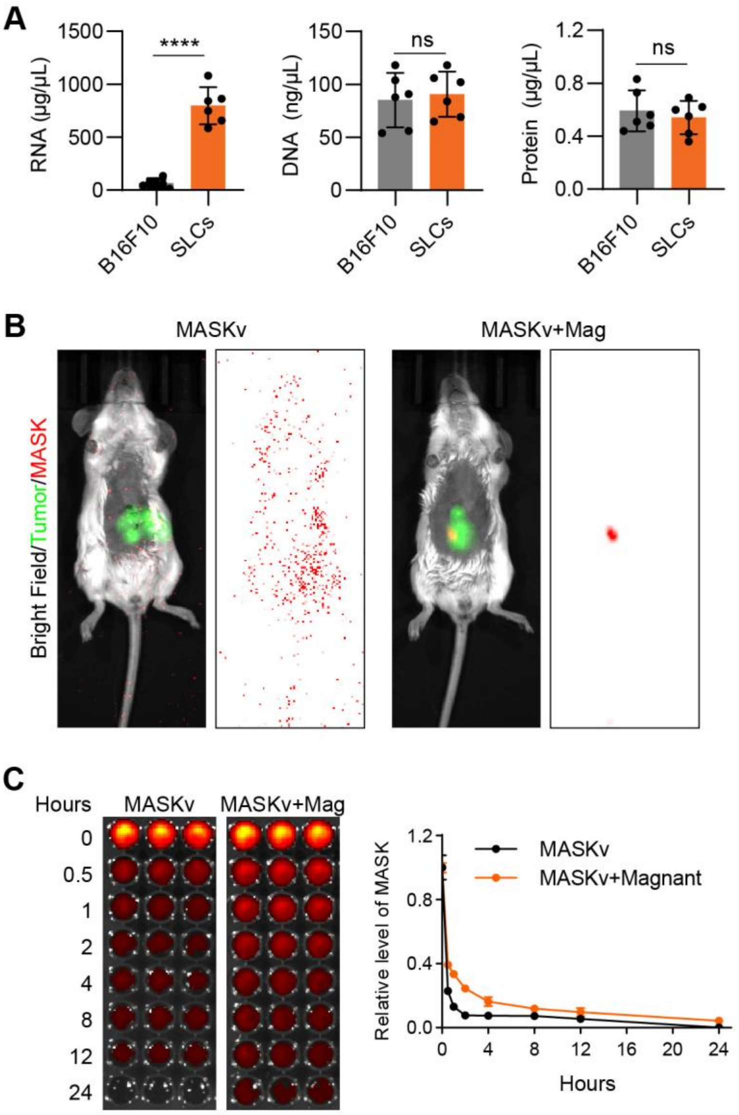

Figure S5. Magnetic Characteristics of SLCs, related to Figure 5.

(A) DNA, RNA and Protein were extracted from B16F10 living cells or SLCs respectively, and the quantitative statistical diagram of each component was obtained after magnetic attraction for 10 minutes (n=6), magnet force = 0.2 T. Data represent analyses of the indicated n mice per group, means ± S.D, and were analyzed by two-tailed unpaired t tests with GraphPad Prism software. ns, not significant, $P > 0.05$; ****$P < 0.0001$. **(B)** IVIS images of the whole body of mice, H22 liver tumor (green) and MASK (red) signal after intravenous injection of DiR-labeled MASKv. **(C)** Images and fluorescence intensities of the blood collected from the mice at indicated time points after intravenous injection of DiR-labeled MASKv.

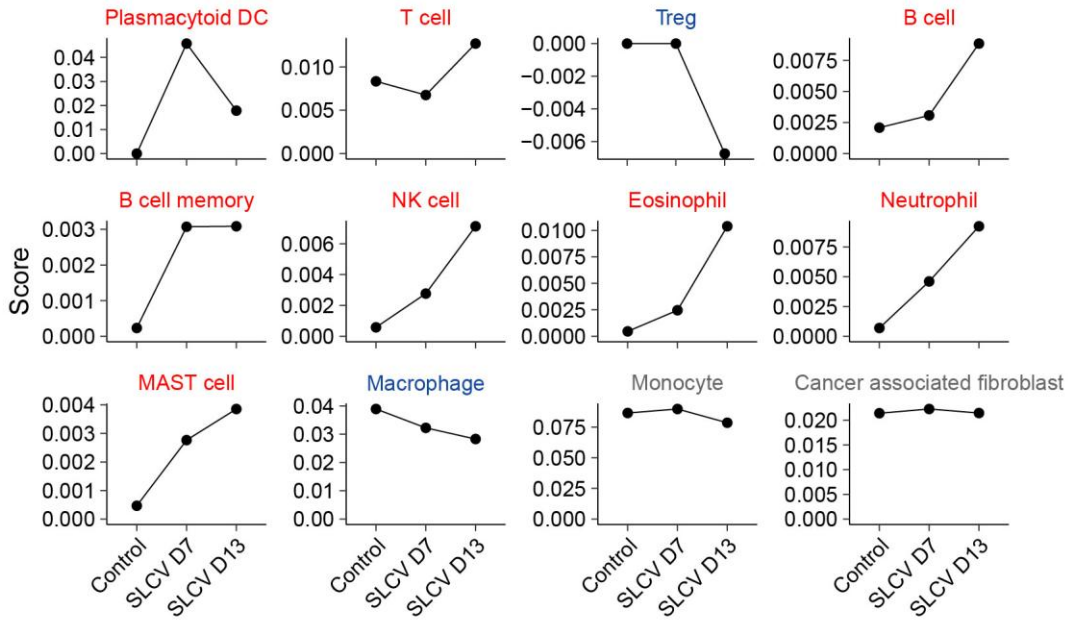

Figure S6. Analysis of SLCV impact on various immune cells using spatial transcriptomics sequencing, related to Figure 6.

Using spatial transcriptomics sequencing data, the mMCP-counter package was employed to calculate the immune cell scores at different time points (Day 7, Day 13) after SLCV treatment. Immune cells marked in red indicate an increased score and activation following SLCV treatment; immune cells marked in blue indicate an increased score and inhibition; and immune cells marked in gray show no significant fluctuation in scores after SLCV treatment.

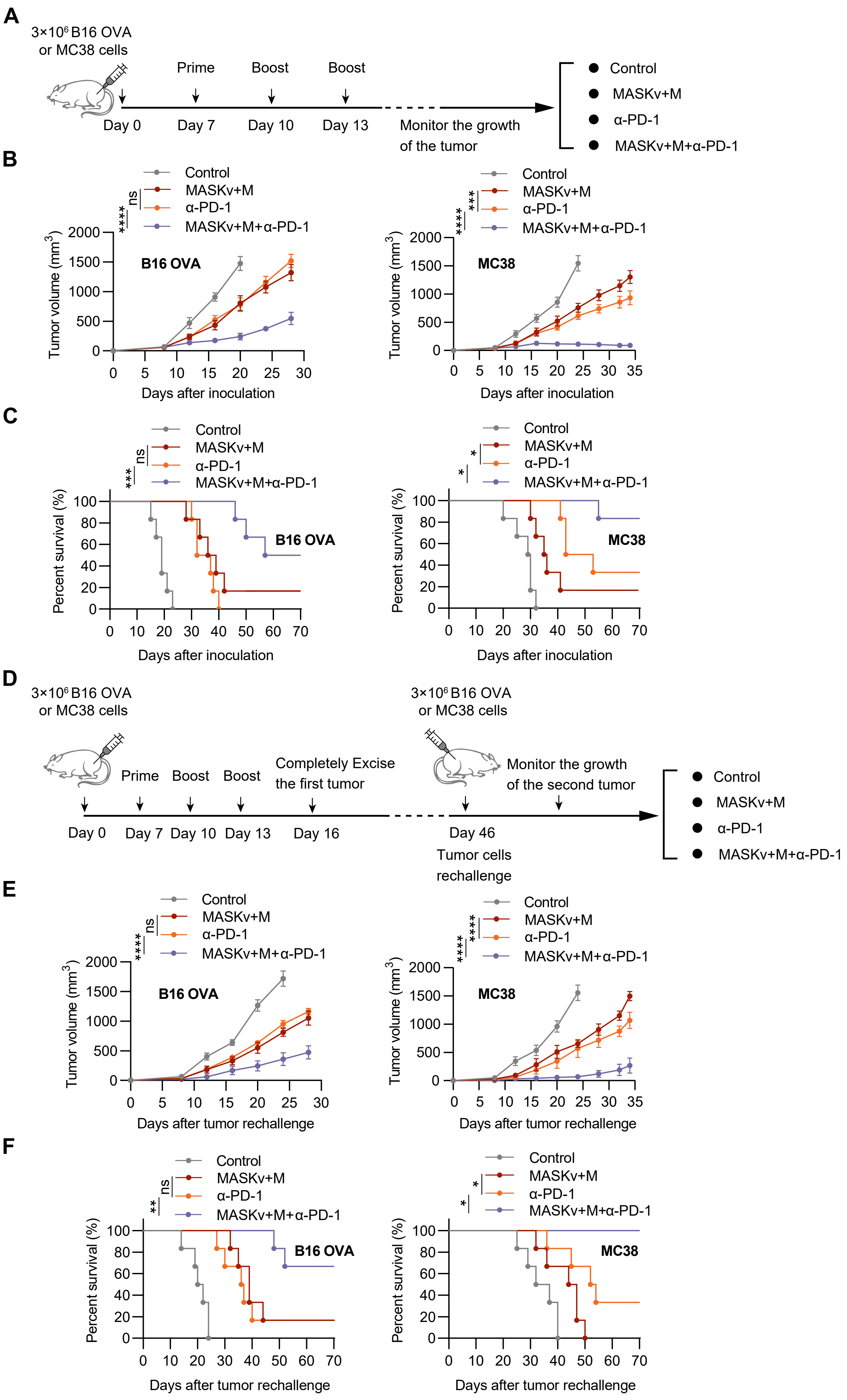
A
3×10⁶ B16 OVA or MC38 cells
Prime
Boost
Boost
Day 0
Day 7
Day 10
Day 13
Monitor the growth of the tumor
Control
MASKv+M
α-PD-1
MASKv+M+α-PD-1
B
B16 OVA
MC38
Tumor volume (mm3)
Days after inoculation
ns
****
***
C
Percent survival (%)
Days after inoculation
*
D
3×10⁶ B16 OVA or MC38 cells
Completely Excise the first tumor
Day 16
Day 46
Tumor cells rechallenge
Monitor the growth of the second tumor
E
Days after tumor rechallenge
F
**

Figure S7. Assessment of combined antitumor efficacy of MASKv and anti-PD-1 antibodies in mice with subcutaneous B16 OVA and MC38 tumors, related to Figure 7.

(A) Schematic of B16 OVA or MC38 tumor model construction and in vivo treatment. **(B)** Tumor volumes were recorded at different time points (n = 6). **(C)** Kaplan–Meier survival curves of the mice of different treatment groups (n=6). **(D)** Schematic representation of B16 OVA or MC38 tumor rechallenge. **(E)** Tumor volumes were recorded at different time points after tumor rechallenge (n = 6). **(F)** Kaplan–Meier survival curves of the mice of different groups (n=6). Data represent analyses of the indicated n mice per group, means ± S.D, and were analyzed by one-way two-sided ANOVA with GraphPad Prism software. ns, not significant, $P > 0.05$; *$P < 0.05$; **$P < 0.01$; ***$P < 0.001$; ****$P < 0.0001$.
